# MoSViT: a lightweight vision transformer framework for efficient disease detection via precision attention mechanism

**DOI:** 10.3389/frai.2025.1498025

**Published:** 2025-03-26

**Authors:** Yuanqi Chen, Aiping Wang, Ziyang Liu, Jie Yue, Enxu Zhang, Fei Li, Ning Zhang

**Affiliations:** ^1^School of Mechanical Engineering, Xijing University, Xi'an, China; ^2^School of Electronic Information, Xijing University, Xi'an, China

**Keywords:** precision attention, maize disease detection, deep learning, MobileViT V2, parallel attention mechanism, few-shot object detection

## Abstract

Maize, a globally essential staple crop, suffers significant yield losses due to diseases. Traditional diagnostic methods are often inefficient and subjective, posing challenges for timely and accurate pest management. This study introduces MoSViT, an innovative classification model leveraging advanced machine learning and computer vision technologies. Built on the MobileViT V2 framework, MoSViT integrates the CLA focus mechanism, DRB module, MoSViT Block, and the LeakyRelu6 activation function to enhance feature extraction accuracy while reducing computational complexity. Trained on a dataset of 3,850 images encompassing Blight, Common Rust, Gray Leaf Spot, and Healthy conditions, MoSViT achieves exceptional performance, with classification accuracy, Precision, Recall, and F1 Score of 98.75%, 98.73%, 98.72%, and 98.72%, respectively. These results surpass leading models such as Swin Transformer V2, DenseNet121, and EfficientNet V2 in both accuracy and parameter efficiency. Additionally, the model's interpretability is enhanced through heatmap analysis, providing insights into its decision-making process. Testing on small sample datasets further demonstrates MoSViT's generalization capability and potential for small-sample detection scenarios.

## 1 Introduction

Food grains constitute an indispensable food source for the burgeoning population in numerous countries (Joseph et al., 2024). Maize is among the world's leading crops (Xu et al., 2023), serving as a vital food source for humanity and being extensively utilized in feed and industrial raw materials (Ji et al., 2024). Its output and quality significantly affect global food security and economic development. Nevertheless, during the growth of maize, pests, and diseases represent the principal threats influencing its yield and quality, which may engender substantial economic losses (Lin et al., 2023). Maize leaf diseases such as maize leaf spot, rust, and dry blight can notably reduce crop yield. The annual loss of maize due to diseases is 6%−10% (Li et al., 2020). Consequently, for farmers lacking professional knowledge (Hao et al., 2020), timely and precise detection and diagnosis of maize leaf diseases is the key to ensuring the healthy growth of crops and evading economic losses for farmers.

Traditionally, the diagnosis of maize leaf diseases has relied on the experience of agricultural experts and visual observation. However, this manual recognition approach has several limitations, such as being time-consuming (Fan et al., 2022), having low efficiency, and being highly subjective (Ahmad et al., 2023). In large-scale farmland settings, expert identification is difficult to implement widely, leading to delays in disease detection and control and increasing crop losses. The continuous evolution of machine vision technology has driven the development of artificial intelligence in agriculture (Li et al., 2023). In recent years, with the rapid advancement of machine learning (Zhang et al., 2018), many researchers have begun to use machine learning methods and computer vision technology for the identification of agricultural diseases and pests (Yuan et al., 2022). Amri et al. (2024) proposed a new deep learning model, MIV-PlantNet, which combines MobileNet, Inception, and VGG architectures to classify diverse plant species in Saudi Arabia. The model achieved 99% accuracy, 96% accuracy, and 98% F1 score, as experimentally proven. Seelwal et al. (2024) systematically reviewed the literature on rice disease recognition from 2008 to 2023, emphasizing the importance of precision-based recognition and advocating hybrid approaches that combine deep learning with machine learning to improve disease recognition efficiency and address agricultural challenges. Gulzar et al. (2023) compared the performance of five deep-learning models in sunflower disease classification and found that EfficientNetB3 performed the best, with an accuracy of 0.979. This indicates that deep learning has significant potential in the early detection and classification of sunflower diseases. Several scholars have conducted studies on the detection of agricultural diseases and pests. Li et al. (2024) improved the ConvNeXt network to detect pepper leaf diseases; Attallah (2023), Kanda et al. (2022), and Shoaib et al. (2022) optimized the classification of tomato leaf diseases using neural networks; Saberi Anari and Kumar (2022), He et al. (2024), and Umamageswari et al. (2023) attempted to detect and categorize various plant leaf diseases, verifying the model's generalizability; Wang et al. (2024) combined a mask autoencoder (MAE) and a Convolutional Block Attention Module (CBAM) to detect 21 leaf diseases and discussed the application of self-supervised learning (SSL) in plant disease recognition. Gulzar (2024) significantly improved the soybean seed classification model by adding five layers to the InceptionV3 architecture and applying techniques such as transfer learning, adaptive learning rate adjustment, and model check pointing. The final model achieved 98.73% accuracy, demonstrating the potential of crop health assessment and management in agricultural technology. These experiments all demonstrate that deep learning technology holds great potential in agricultural pest detection.

Recent studies have also focused on the classification of maize leaf diseases using deep learning techniques. Alkanan and Gulzar (2024) used artificial intelligence technology to identify and classify maize diseases. By improving the MobileNetV2 model and adding an outer layer, combined with various optimization techniques, they achieved a classification accuracy of about 96%, performing well compared to advanced models. Haque et al. (2022b) trained the Inception v3 model with maize leaf disease images collected by their team, achieving an average recall rate of 95.96%, although the model's accuracy was unsatisfactory. Qian et al. (2022) improved the attention mechanism, resulting in an average recall rate of 98%. Amin et al. (2022) proposed a composite model scaling technique, designing a fusion of coefficients from two models to enhance detection accuracy, achieving a final detection accuracy of 98.56%. Li et al. (2022) designed a filter layer to preprocess images, reducing noise interference and replacing the activation function to minimize overfitting, reaching an average accuracy of 97.96%. Pan et al. (2022) combined the softmax loss function with the GoogleNet model to detect northern maize leaf blight, achieving an impressive detection accuracy of 99.94%. However, these studies have certain limitations. Although detection accuracy has steadily improved, the large size of the models makes them impractical for real-time detection in resource-limited environments. Acknowledging this issue, researchers have started to focus on creating smaller, faster models. Haque et al. (2022a) introduced modified Inception modules to develop a lightweight network model for detecting maize leaf blight, offering a single detection target but providing ideas for further improvements. Fan and Guan (2023) designed a maize disease recognition system based on a pre-trained VGG16 model, applying transfer learning to improve performance. The lightweight VGNet model occupied only 79.5 MB and took 75.21 seconds to test 230 images, demonstrating a good recall rate with sufficient data. Bi et al. (2023) improved MobileNetV3 by adding bias loss functions and an ECA attention mechanism, ensuring better performance while maintaining a small model size. Zhou et al. (2024) chose the compact ShuffleNetV2 model as a base for improvements, replacing deep convolution layers with a max-pooling layer for downsampling, extracting key features, and reducing overfitting. This resulted in an accuracy of 98.40% with a model size of just 1.56 MB, offering a novel approach for model enhancement. However, reducing model size often compromises detection accuracy, and real-world environments with significant noise and complexity (Masood et al., 2023; Craze et al., 2022; Ma et al., 2022) make traditional deep-learning models less effective. To address this, Zeng et al. (2022) adjusted their model by incorporating a joint focal loss function to tackle data issues, achieving an average recognition accuracy of 92.9%, which was still unsatisfactory. Cai et al. (2023) used EfficientNet, integrating an adaptive fusion module to merge image information at different scales and designing a coordinate attention module based on full convolution to reduce background interference. With 3.44 million model parameters and 339.74 million FLOPs, the recognition accuracy remained promising. Chen et al. (2022) developed the DFCANet model, incorporating a dual feature fusion module and a coordinate attention-based downsampling module to reduce information loss by expanding feature channels and using deep convolution, aiming to mitigate noise interference with a more structured model design. Albahli and Masood (2022) designed the Efficient Attention Network (EANet) to identify multiple maize diseases, introducing a spatial channel attention mechanism. They trained the EANet model using focal loss to address class imbalance issues and applied transfer learning to enhance generalization, achieving an overall experimental accuracy of 99.89%. Nevertheless, current model improvements generally lack a scientifically explainable foundation, which may result in a disconnect between the methods and their objectives, limiting future development. It seems that after introducing new components, researchers are attempting to mix new “spices” with old “recipes.” While the results may improve, how the changes in “flavor” align with the desired outcomes remains unclear.

Therefore, this paper makes the following main contributions:

1. It proposes a new perspective for classification models: the model should prioritize precise feature extraction rather than comprehensively focusing on all features. Traditional models often have broad coverage, frequently capturing irrelevant features or leading to misjudgments. By concentrating on key features, the model can maintain high classification accuracy while improving learning efficiency.
2. Significant improvements have been made to the MobileViT V2 framework, introducing a new attention mechanism, two additional modules, and a new activation function to support the proposed point attention theory.
3. The model achieves an average accuracy of 98.75% and a peak accuracy of 99.19%. It also performs well on datasets with smaller sample sizes.

## 2 Data and methods

### 2.1 Data set construction

**Figure 1:**
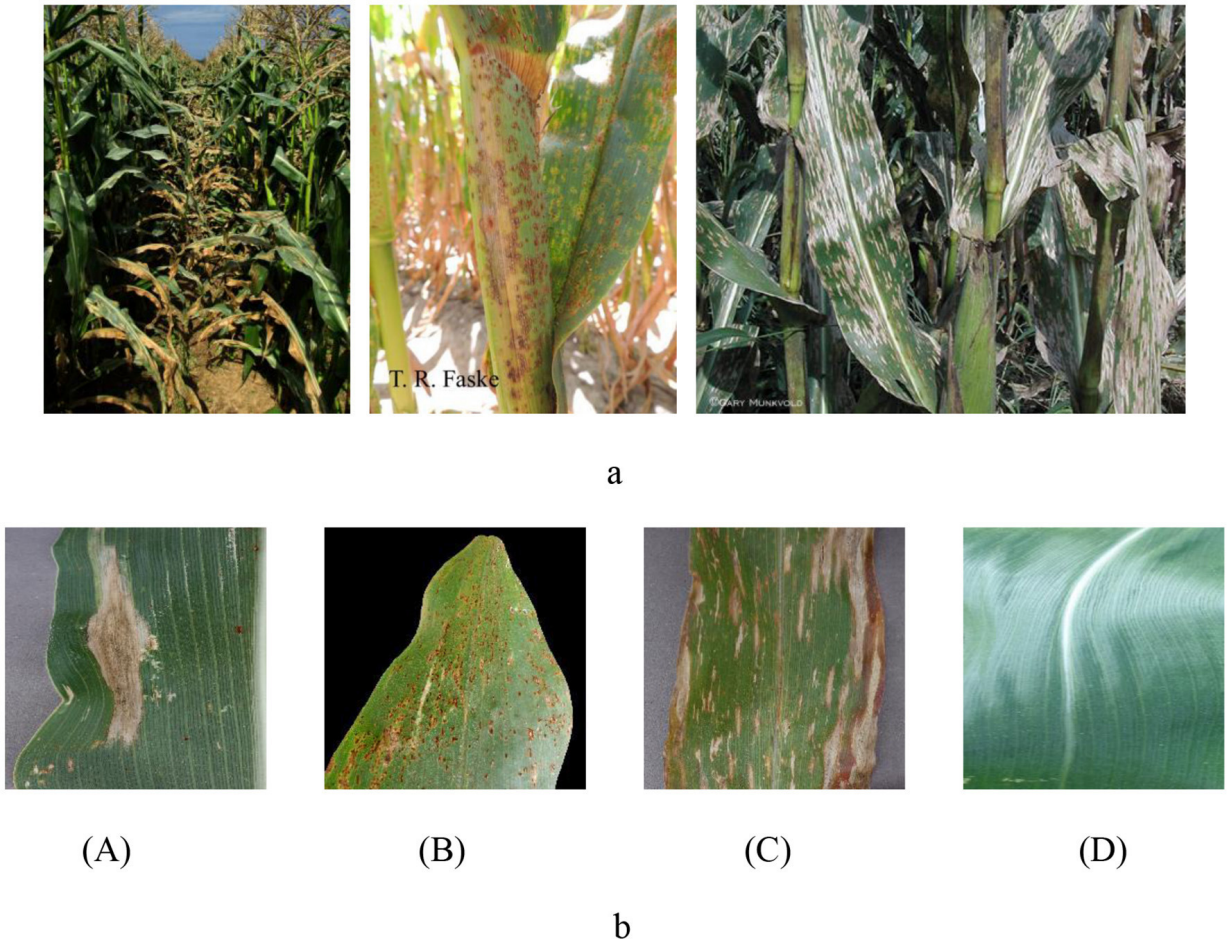
**(A)** Non-conforming maize leaf image. **(B)** Maize leaf image. A: Blight; B: Common Rust; C: Gray Leaf Spot; D: Healthy.

In this study, we used a carefully constructed maize leaf image dataset from the PlantVillage and PlantDoc datasets (Singh et al., 2019; Kusumo et al., 2018). These datasets are widely recognized as an important public resource in the field of plant pathology, providing a robust and reliable repository for our research. However, in each category of the source data set, there are some images whose detection targets are not clear or whose resolution reaches the threshold, as shown in Figure 1A. After checking the data set and removing images that did not meet the detection criteria, we successfully reconstructed a dataset containing four major categories of rusty leaves, gray mottled leaves, withered leaves, and healthy leaves (Yang et al., 2024). A visual representation of each category is shown in Figure 1B.

### 2.2 Data balancing

**Figure 2:**
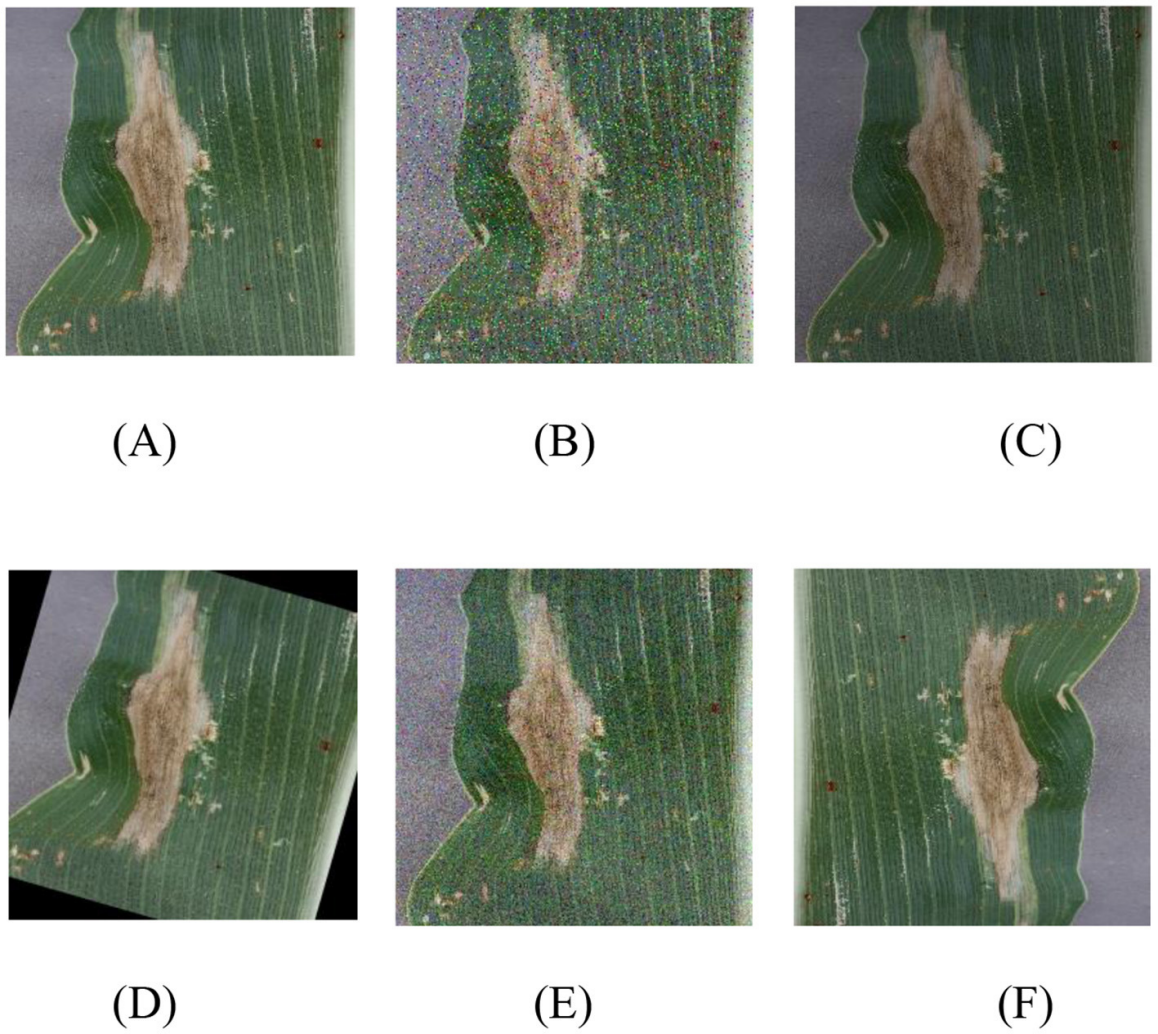
Image balancing process image, where **(A)** original image; **(B)** Salt and pepper noise; **(C)** random brightness; **(D)** Random Angle rotation; **(E)** Gaussian blur; **(F)** 180° rotation.

The number of maize leaves in the Gray_Leaf_Spot category is significantly smaller compared to other categories, which could negatively impact the accuracy and stability of the model's classification (Yang et al., 2024; Ayoub et al., 2023). To address the issue of class imbalance and enhance the model's generalization capability, we implemented a series of data augmentation techniques to balance the dataset. These techniques not only increase the volume of data but also simulate various real-world image variations, thereby improving the model's robustness. Specifically, we applied the following processing methods to each image: random brightness adjustment, Gaussian noise addition, salt-and-pepper noise introduction, random angle rotation, and image flipping. A processed example is shown in Figure 2. By employing these data augmentation techniques, we not only balanced the sample sizes across different categories but also significantly expanded the size and diversity of the training set, as demonstrated in Table 1 before and after data balancing. This approach effectively reduces the risk of model overfitting and enhances the model's adaptability to a range of real-world scenarios.

**Table 1:** Quantity of samples before and after equilibrium.

**Classes**	**Original quantity**	**Balance quantity**
Blight	983	5,898
Common_Rust	1,192	7,152
Gray_Leaf_Spot	513	3,087
Healthy	1,162	6,972

### 2.3 MobileViT V2 network model

**Figure 3:**
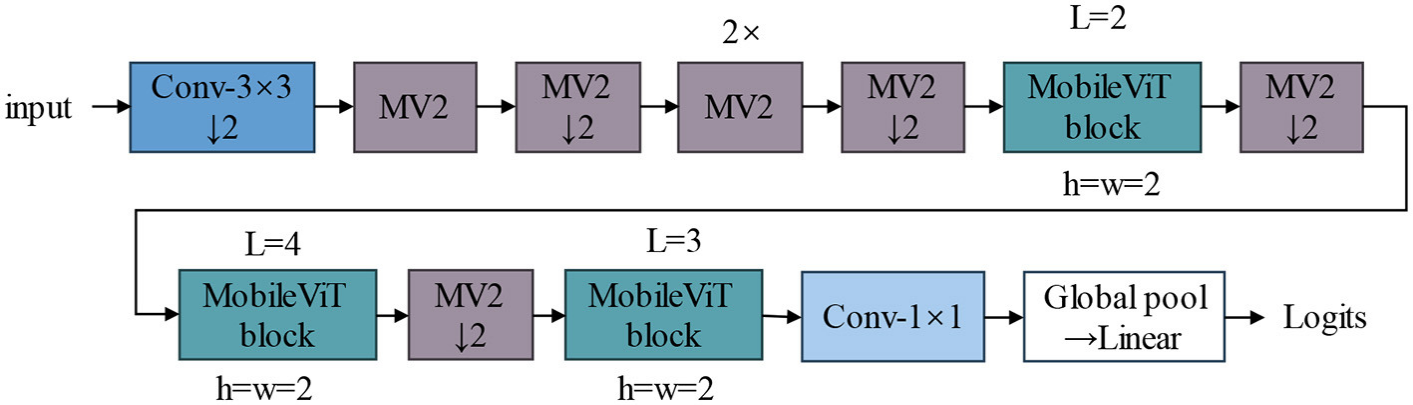
MobileViT V2 network structure diagram, MV2: MobileNet V2 block.

In this study, we have made enhancements based on the MobileViT V2 model (Mehta and Rastegari, 2022). MobileViT V2 is a highly efficient vision transformer model designed to deliver exceptional performance and computational efficiency on mobile devices. The model integrates the strengths of convolutional neural networks and vision transformers, effectively extracting both local, and global features through its flexible module design. MobileViT V2 excels in classification tasks, providing highly accurate results while maintaining low computational overhead. Its architecture features innovative convolution and transformer modules that can handle complex visual tasks while optimizing computation and memory usage across various devices. The structure diagram of MobileViT V2, shown in Figure 3, illustrates the configuration and interconnection of its modules.

### 2.4 Backbone network

**Figure 4:**
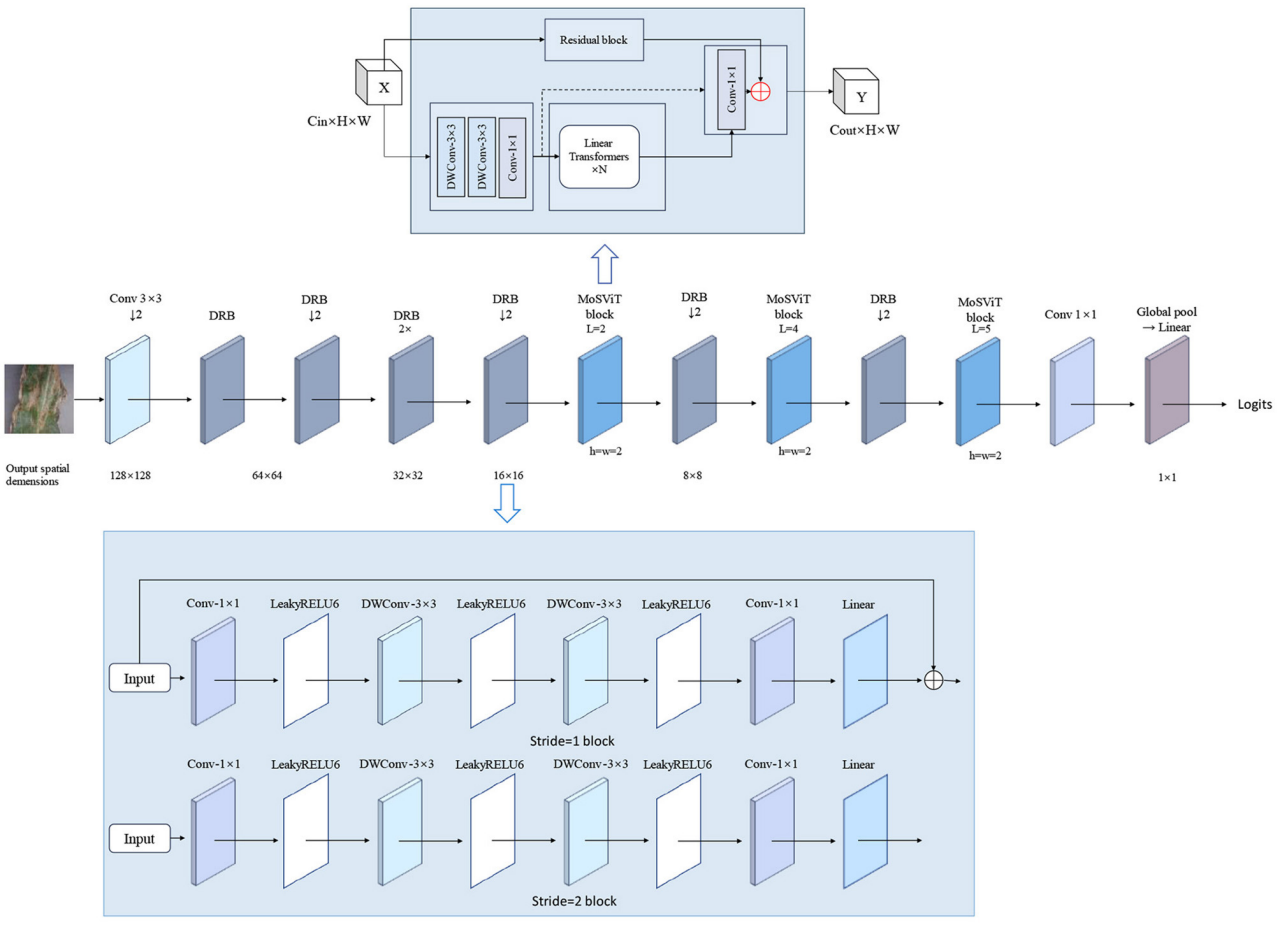
CLA-MoSViT backbone network structure.

The CLA-MoSVIT model proposed in this paper is employed for the identification of maize leaf diseases and is constituted by the DRB Block, MoSViT Block, Transformer Block, and CLA attention mechanism. During model training, the DRB Block, functioning as a CNN module, is utilized to downsample the input images and initially extract their features. Meanwhile, the MoSViT Block encompasses multiple CNN and Transformer modules for the profound extraction of the input image features. The DRB Block comprises two 1 × 1 convolutional kernels and two DW 3 × 3 convolutional kernels, which mainly handle the input image to reduce its size and extract features through successive 3 × 3 convolutional kernels. On one hand, the MoSViT Block initially extracts features through consecutive DW 3 × 3 convolutional kernels and then transmits the output to both the 1 × 1 convolutional kernel and the Transformer block simultaneously. The output of the Transformer block is used as the input to the 1 × 1 convolutional kernel to adjust its shape. On the other hand, the initial input is transferred to the depth-separable residual block for feature extraction. Ultimately, the addition of the outputs from the three parts is regarded as the final output. The MoSViT Block achieves efficient feature extraction by combining CNN, Transformer, and dense connection approaches, thereby obtaining higher detection accuracy. Its structure is depicted in Figure 4 and Table 2.

**Table 2:** Structure of CLA-MoSViT model.

**Layer**	**Output size**	**Out stride**	**Repeat**
Input	256 × 256	1	-
Conv-3 × 3, ↓2 DRB	128 × 128	2	1 1
DRB, ↓2 DRB	64 × 64	3	1 2
DRB, ↓2 MoSViT Block	32 × 32	8	1 2
DRB, ↓2 MoSViT Block	16 × 16	16	1 2
DRB, ↓2 MoSViT Block Conv-1 × 1	8 × 8	32	1 1 1
Global average pooling	1 × 1	256	1

Figure 6 shows the DRB module, and Figure 9B shows the structure of the MoSViT Block.

### 2.5 CLA attention mechanism

**Figure 5:**
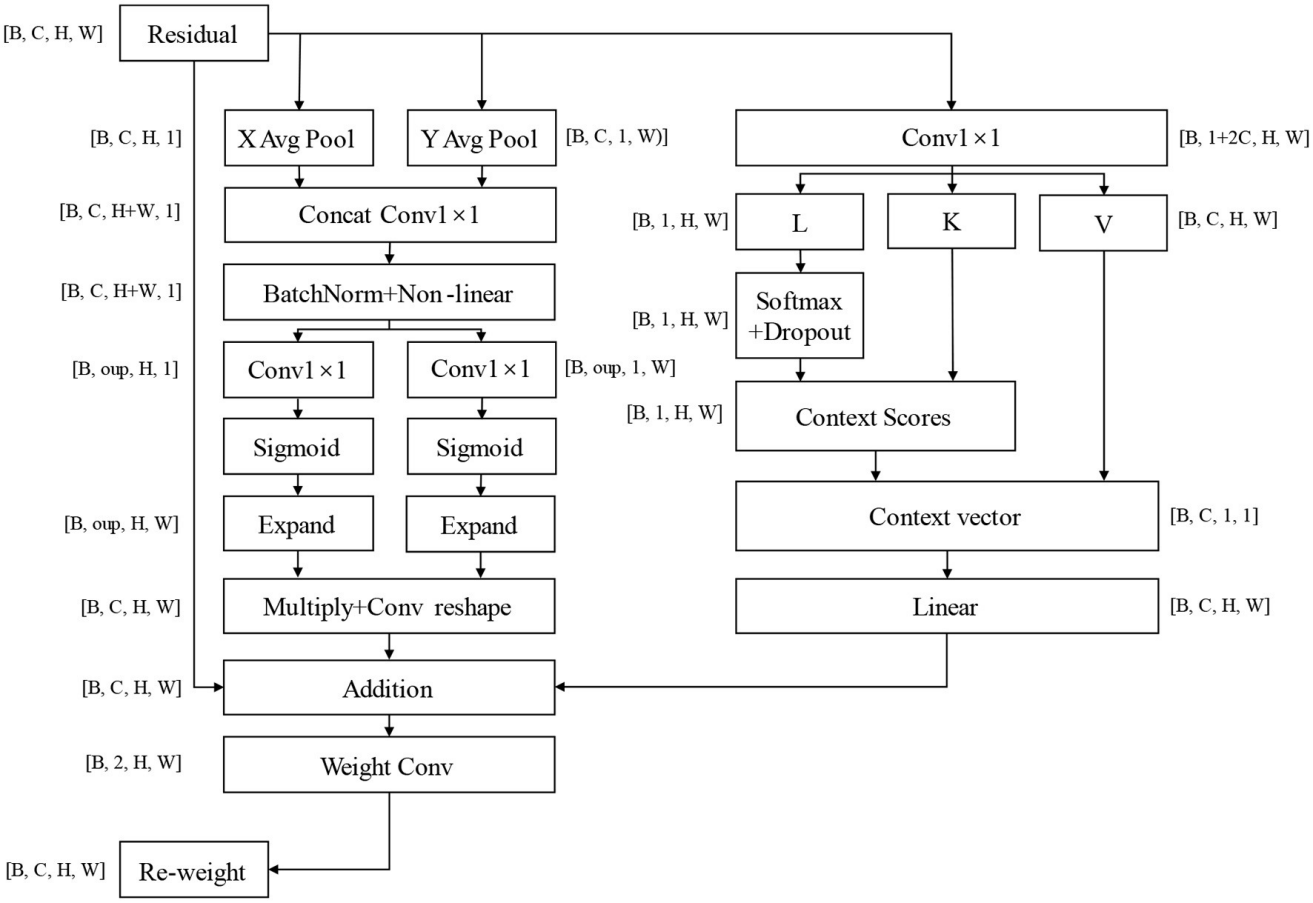
CLA attention mechanism structure, oup: abbreviation of output channels, refers to the convolutional layer or the number of characteristic channels output of attention mechanism; Multiply means a multiplication operation at the element level; The Expand operation is used to expand the tensor repeatedly in some dimensions; Context Scores are obtained through softmax operation on query; Context Vector is obtained by weighted summing Context Scores with key. It captures global context information for input features.

Contemporary feature extraction models aim to achieve broader coverage of feature spaces. However, this expanded capture scope inevitably leads to increased probability of irrelevant feature inclusion and more complex model architectures, which consequently compromise the requirements for accurate recognition and rapid response in complex environments. Therefore, we contend that precise feature identification and classification are paramount for classification models. Based on this theoretical framework, we have developed the CLA (Channel-Location Adaptive) attention mechanism, which enhances focus while maintaining global information capture. The architectural configuration of this attention mechanism is illustrated in Figure 5.

#### 2.5.1 Channel attention module

The core concept of the CLA mechanism lies in aggregating features along horizontal and vertical directions to generate position-sensitive attention maps. Specifically, it employs two adaptive average pooling operations to compress the feature map along the height and width dimensions respectively, capturing global contextual information in horizontal and vertical orientations. The compressed features undergo convolution and non-linear activation operations to generate channel-level attention weights. These weights are then applied to the original feature map to achieve spatially sensitive feature enhancement. This design enables the model to precisely identify and emphasize the most discriminative regions in images rather than uniformly attending to the entire image. By accurately localizing and enhancing critical spatial features, the CLA mechanism significantly improves the model's ability to capture essential information while reducing interference from irrelevant features, thereby enhancing classification accuracy and model robustness. The module weight calculation formula is shown in Equation 1.


(1) $$\begin{array}{l} A\text{\_}h=Sigmoid\left(Convlxl\left(Z\text{\_}h\right)\right)\\ A\text{\_}w=Sigmoid\left(Convlxl\left(Z\text{\_}w\right)\right) \end{array}$$

In the formula, Z_h and Z_w are the feature representations obtained by convolution and activation of the pooled feature graph, and A_h and A_w are the spatial attention weights calculated in the height and width directions, representing the importance of each position in the height and width directions respectively. The application formula of feature weighting is shown in Equation 2.


(2) $$\begin{array}{l} Output=X*Expand\left(A\text{\_}h\right)*Expand\left(A\text{\_}w\right) \end{array}$$

Where Expand(A_h) and Expand(A_w) are to expand the attention weight to the same spatial size as the input feature plot for pixel-by-pixel multiplication.

In this way, the model can highlight important spatial locations while suppressing irrelevant areas, allowing the model to focus on capturing the right key features.

#### 2.5.2 Self-attention module

The self-attention module is designed to efficiently capture intrinsic relationships between features. Through 1 × 1 convolution operations, it generates corresponding query, key, and value vectors. This method enables independent evaluation of feature importance distribution at each spatial position, with attention scores reflecting the relative importance of different spatial locations calculated using the softmax function. By multiplying these attention scores with the value vectors and performing weighted summation, the model can focus on the most relevant feature information. Finally, another 1 × 1 convolution operation maps the enhanced features back to the original feature space. The design philosophy of this module aligns closely with the core thesis of this paper, effectively identifying and enhancing the most discriminative local features while maintaining computational efficiency. This lightweight design allows the model to process information rapidly, concentrating computational resources on critical feature regions essential for classification tasks rather than wasting them on potentially irrelevant areas. The context vector calculation formula is presented in Equation 3.


(3) $$\begin{array}{r} Context\_vector=Sum(K*Attention\_scores,dim=-1,\\ keepdim=true) \end{array}$$

In the formula, *K* represents the key vector, and *Attention*_*scores* are the attention scores. The operation *Sum* (*K* * *Attention*_*scores, dim* = −1, *keepdim* = *True*) calculates the context vector by weighting and averaging the key vectors, thereby obtaining a weighted feature representation. This computation enables the model to concentrate on the most pertinent features via attention mechanisms while taking into account global information. The design effectively balances wider feature capture with precision.

#### 2.5.3 Union module

The joint attention module adopts a dual-branch parallel architecture: the channel attention branch specializes in precise spatial feature localization, while the self-attention branch focuses on modeling interrelationships between local features. This design demonstrates our paper's core thesis that the integration of parallel complementary attention mechanisms with residual structures enables comprehensive and accurate feature extraction. Notably, the module's dynamic weight allocation mechanism allows automatic adjustment of attention strategies based on input characteristics—enhancing the channel attention branch for critical region localization while prioritizing the self-attention branch when analyzing complex feature relationships. This adaptive capability not only improves the model's discriminative feature capture capacity but also effectively reduces misclassification risks by suppressing interference from irrelevant information. The mathematical formulation of the weight generation process is detailed in Equation 4.


(4) $$\begin{array}{l} Weights=Normalize\left(Convlxl\left(Y\text{\_}combined\right)\right) \end{array}$$

In the formula, Normalize (Weights, p = 1, dim = 1) the weights are normalized L1 to make their sum equal to 1, ensuring that the weighting operation is balanced.

The final output is shown in Equation 5.


(5) $$\begin{array}{r} Output=Weights\left[:,0:1,:,:\right]*Y\text{\_}coord+Weights\left[:,1:2,:,:\right]\\ {}*Y\text{\_}linear \end{array}$$

Where Weights[:, 0:1,:,:] and Weights[:, 1:2,:,:] are the coefficients for weighting the feature graph.

This dynamic weighting mechanism allows the model to adaptively adjust the importance of different attention mechanisms based on inputs. Enhance the model's ability to reduce error features and environmental impacts.

### 2.6 DRB module

**Figure 6:**
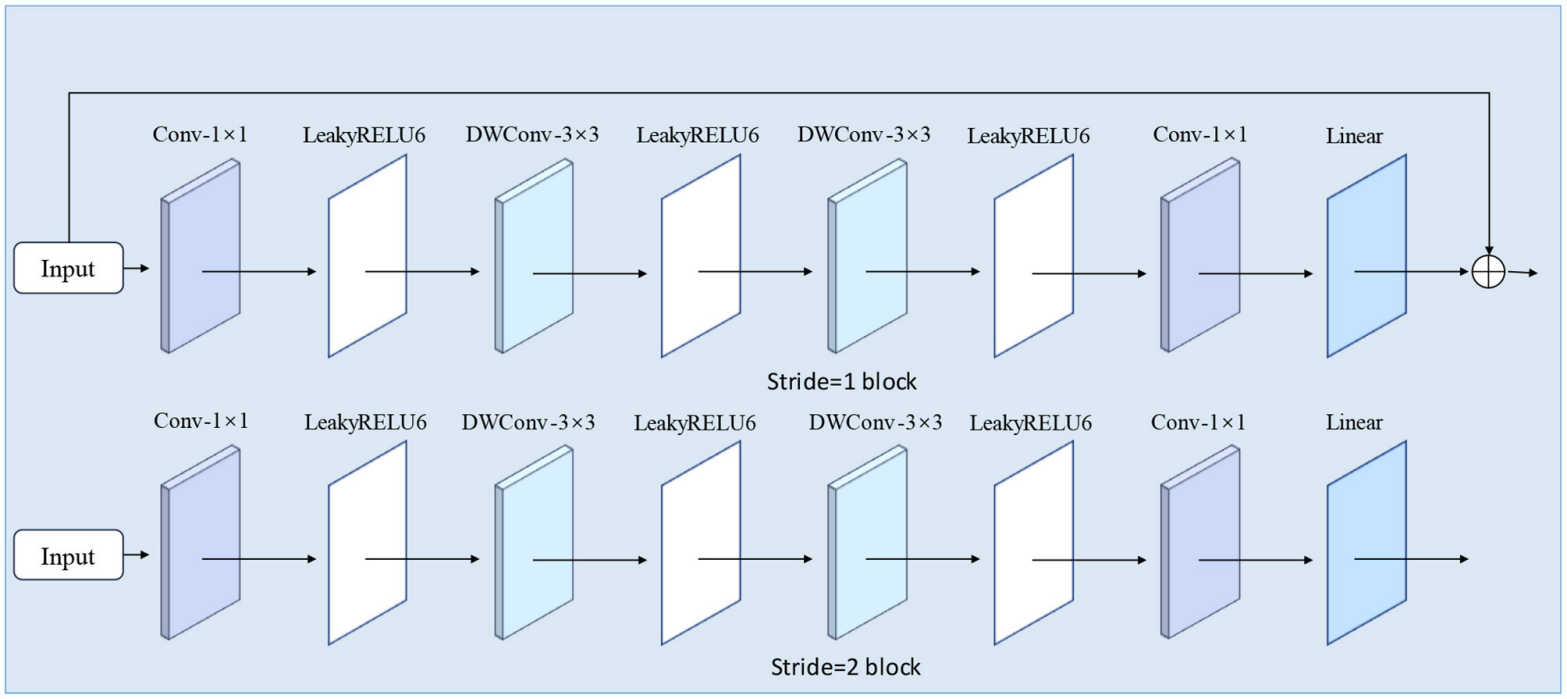
DRB module structure.

In this study, we have made structural enhancements to the inverted bottleneck module in MobileViT V2 to improve both feature extraction capabilities and overall model performance. The modified DRB module structure is illustrated in Figure 6. The original inverted bottleneck module in the MobileViT V2 model employs a specific sequence of convolutional layers. We have redesigned this structure by introducing additional convolution operations and incorporating residual connections.

**Figure 7:**
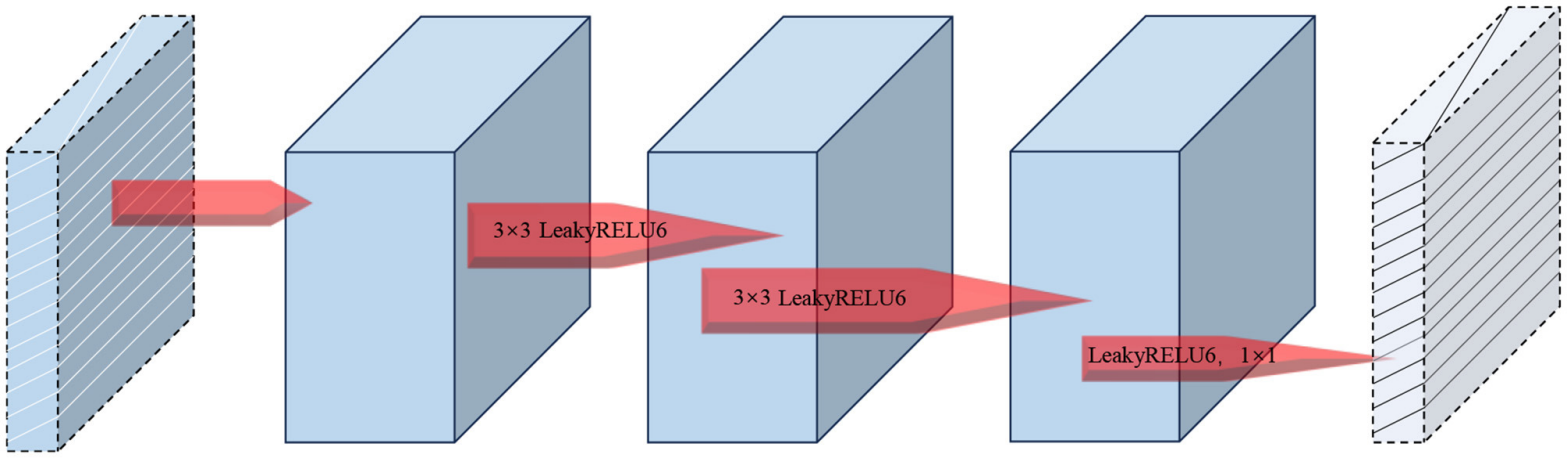
Information collection process of the DRB module.

Our improvements focus on enhancing the model's ability to capture local features and mitigating the gradient vanishing problem in deep networks, thereby facilitating more effective model training. Specifically, we have added a depthwise separable convolution layer to enhance the model's local feature extraction. Additionally, we have included a residual connection that directly adds the input to the module's output. This modification not only boosts the model's feature expression capability but also helps preserve and utilize the original input information, potentially enhancing the quality of feature representation. To prevent overfitting in the DRB module, we have used LeakyReLU6 as the activation function. This choice helps maintain a balance between non-linearity and computational efficiency. The module's information collection process is depicted in Figure 7.

### 2.7 Activation function

We have improved the activation function, and in the original model, the activation function is replaced by the H-Swish function, which is an approximate version of the Swish function that is more computationally efficient. The mathematical expression for H-Swish is shown in Equation 6.


(6) $$\begin{array}{l} h-swish\left(x\right)=x\frac{ReLU6\left(x+3\right)}{6} \end{array}$$

ReLU6 is a variant of the ReLU function that limits its output value to a maximum of 6.

There are several key reasons for opting for the H-Swish function. First, H-Swish generally outperforms traditional ReLU and ReLU6 functions in many tasks, particularly in deeper networks. It offers the advantage of non-linear activation while also maintaining a robust gradient flow. Second, although H-Swish is more complex than the simple ReLU, it is more efficient than the original Swish function, making it especially well-suited for mobile-friendly models. The smoothness of the H-Swish function aids in the optimization process, potentially leading to better convergence. Thus, H-Swish strikes a favorable balance between model performance and efficiency when deployed on mobile devices.

Additionally, we have introduced a novel activation function, LeakyReLU6, in the DRB module. The calculation formula for LeakyReLU6 is provided in Equation 7.


(7) $$\begin{array}{l} LeakyReLU6\left(x\right)=min\left(max\left(ax,x\right),6\right) \end{array}$$

**Figure 8:**
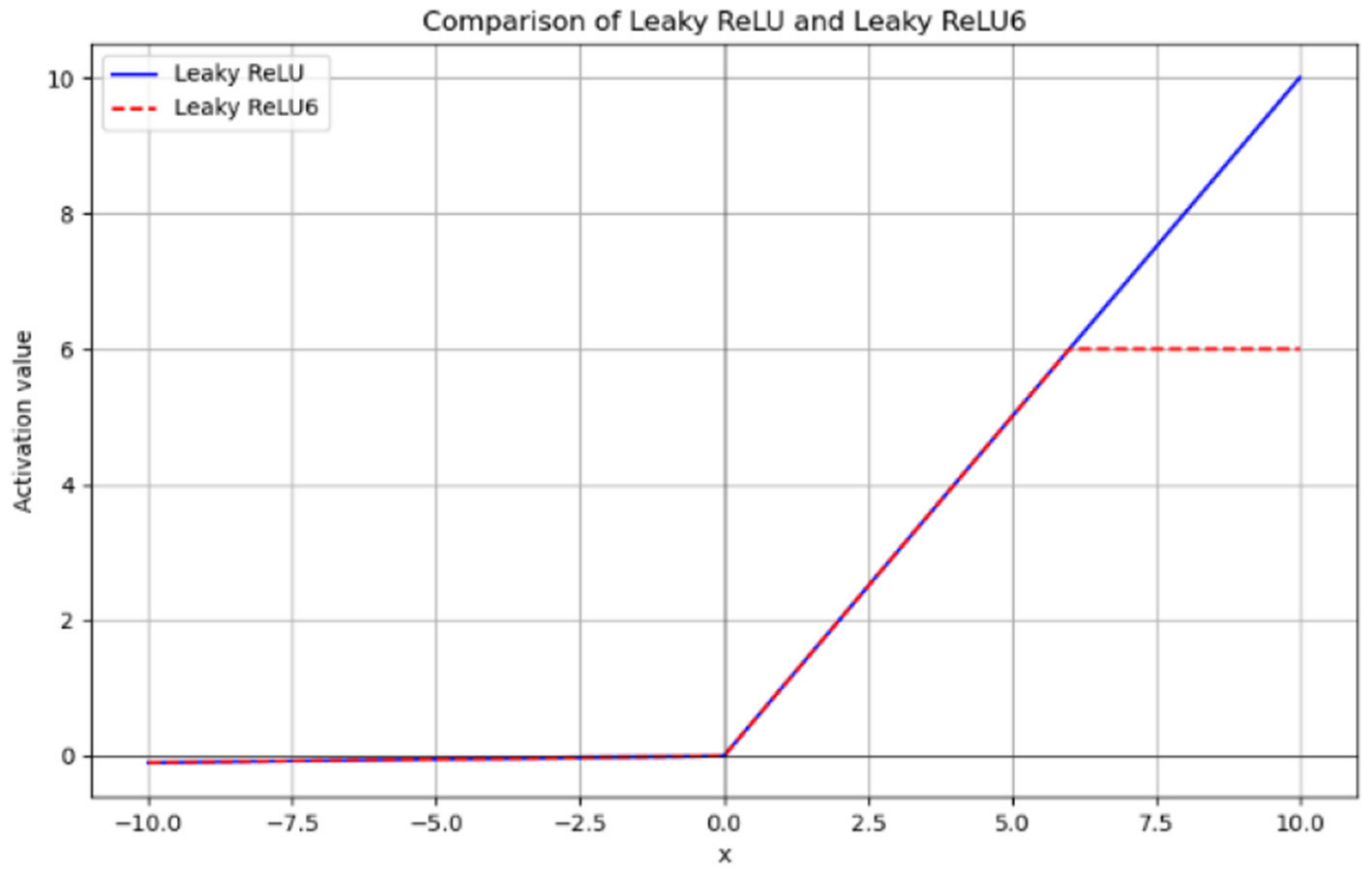
Function image of LeakyReLU6.

Where x is the input value and 6 is the upper bound of the activation function. The function image for LeakyReLU6 is shown in Figure 8.

By limiting the maximum range of activation values, LeakyReLU6 can effectively avoid excessive activation values in low-precision computing environments. This helps reduce the inaccuracy of numerical representation and improve the stability of calculations. At the same time, it retains the advantage of LeakyReLU, which allows negative values to pass, thus alleviating the gradient disappearance problem and mitigating the “dying ReLU” issue to some extent. The introduction of a maximum limit in LeakyReLU6 also effectively reduces the risk of overfitting the model by preventing it from over-relying on certain features during training.

### 2.8 MoSViT block

**Figure 9:**
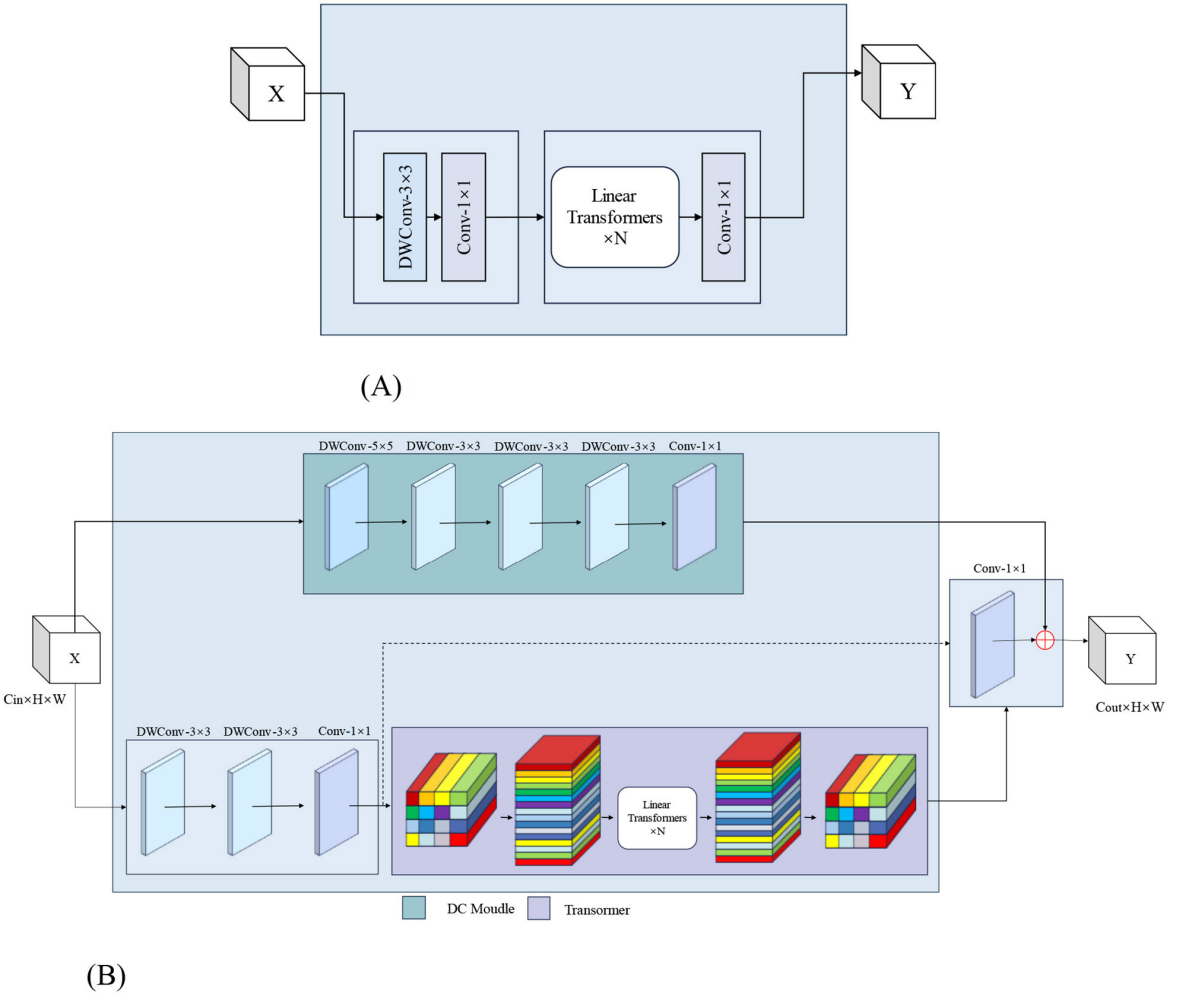
**(A)** Structure of the MobileViT Block module. **(B)** MoSViT Block module structure, DC module: dense link depth separable convolutional module.

The MoSViT Block is a crucial component of the model, undergoing numerous enhancements based on the MobileViT Block module. Figure 9A illustrates the structure of the original MobileViT Block module, while Figure 9B showcases the refined MoSViT Block module.

In the improved module, the input initially splits into two parallel processing streams. The first stream starts with two consecutive 3 × 3 depthwise convolution (DW) layers, designed to extract spatial features while maintaining computational efficiency. Afterward, the processing splits into two sub-streams: one sub-stream focuses on channel fusion and dimensionality reduction through a 1 × 1 convolution, while the other introduces a Linear Transformer block. The output from the Transformer is then directed into a 1 × 1 convolution to reshape the features and align them with the primary stream.

Simultaneously, the module's second primary processing stream directly feeds the raw input into the DC module. This DC module consists of multiple depthwise separable convolutional layers, where the input to each layer is a concatenation of the outputs from all preceding layers. Finally, the outputs from these three distinct processing streams—the 1 × 1 convolution, the DC module, and the Transformer path—are integrated through an element-wise addition operation to produce the module's final output.

## 3 Experiment and result analysis

### 3.1 Experimental environment

In this study, we conducted a series of experiments to compare classification models using the PyTorch deep learning framework on a Windows operating system. The image input size was set to 224 × 224. Since the model was observed to converge within 100 epochs, we set the number of epochs to 100. The specific parameters of the experimental setup are detailed in Table 3.

**Table 3:** Experimental parameters.

**Accessories**	**Parameter**
CPU	12th Gen Intel(R) Core(TM) i7-12700 2.10 GHz
Random access memory (RAM)	16.0 GB
Vm(G)	12
Language	Python3.9.12
DF	Pytorch1.13.1
CUDA	11.6
Epoch	100

Vm, Video memory; Df, Development framework.

The learning rate, a crucial factor in the training process, was tested at values of 0.001, 0.0001, and 0.00001. A learning rate that is too high may prevent the model from converging, while one that is too low can significantly slow down the training process. We also experimented with batch sizes of 8, 16, and 32 to determine the optimal setting. Different combinations of learning rates and batch sizes can lead to changes in detection accuracy. The results are presented in Table 4.

**Table 4:** Classification model performance based on learning rate and batch size.

**Learning rate**	**Batch size**	**Converged epoch**	**Accuracy**
0.001	8	20	95.89%
0.0001	8	20	94.34%
0.00001	8	20	94.77%
0.001	16	20	96.59%
0.0001	16	30	96.55%
0.00001	16	20	97.60%
0.001	32	20	98.06%
0.0001	32	20	98.47%
0.00001	32	20	98.75%

### 3.2 Evaluation index

In this experiment, several key metrics were used to evaluate model performance. Accuracy measures the proportion of samples predicted correctly by the model out of all samples and is the most intuitive performance indicator. Precision refers to the proportion of samples predicted by the model to be positive that are positive, which is particularly important for dealing with imbalanced datasets. The recall represents the proportion of actual positive samples that the model successfully predicts, reflecting the model's ability to capture positive samples. The F1-Score is the harmonic mean of precision and recall, taking both metrics into account and serving as an important indicator for evaluating the overall performance of the model. Finally, the number of parameters in the model directly affects its storage requirements and computational complexity. Models with fewer parameters occupy less memory and storage space and have faster computation speeds. This is especially important in resource-constrained environments or scenarios requiring efficient computations. The detailed process for calculating these evaluation metrics is shown in Table 5.

**Table 5:** Calculation formula of evaluation index.

**Index**	**Formula**	**Significance**
Accuracy	$Accuracy=\frac{TP+TN}{TP+TN+FP+FN}$	Correctly estimate the number as a percentage of the total.
Precision	$Precision=\frac{Tp_{i}}{Tp_{i}+FP_{i}}$	The probability that all samples predicted by the model to be positive examples are positive examples
Recall	$Recall=\frac{Tp_{i}}{Tp_{i}+FN_{i}}$	The probability that the model predicts correctly in all positive cases
F1-Score	$F1=2\times \frac{Recall\times Precision}{Recall+Precision}$	F1 score takes into account both recall and accuracy
Number of parameters	-	Measure the complexity of the model

TN (True Negative): the negative example is correctly judged as a negative example. TP (True Positive): indicates that a positive example is correctly judged as a positive example. FN (False Negative): indicates that a positive example is incorrectly judged as a negative example. FP (False Positive): indicates that a negative example is incorrectly judged as a positive example.

### 3.3 Result analysis

#### 3.3.1 Ablation experiment

To verify the effectiveness of the CLA attention mechanism, DRB, and MoSViT Block modules in the CLA-MoSViT algorithm, we conducted a series of ablation experiments. These experiments aimed to evaluate the specific contributions of the CLA attention mechanism and the dense linking modules to model performance. We set up five sets of experiments: the baseline model used the standard MobileViT V2, while the other four sets individually incorporated the CLA attention mechanism, the DRB module, the MoSViT Block module, and a combination of all three modules, respectively.

As shown in Table 6, the experimental results indicate that the accuracy of the original model is 95.33%. After integrating the CLA attention mechanism, the accuracy increased to 95.80%. Incorporating the DRB module alone raised the accuracy to 96.50%. The MoSViT Block module, when used individually, improved the accuracy to 97.20%. Finally, when all three modules (CLA, DRB, and MoSViT Block) were combined, the accuracy increased to 98.75%.

**Table 6:** Results of the ablation experiment.

**Evaluation index**	**Original model (%)**	**Original model +CLA (%)**	**Original model +DRB (%)**	**Original model +MoSViT Block (%)**	**Original model +CLA+ DRB+ MoSViT Block (%)**
Accuracy	95.33	96.24	96.03	97.28	98.75
Precision	95.80	96.21	96.08	97.29	98.73
Recall	95.79	96.24	96.04	97.26	98.72
F1 Score	95.72	96.11	96.05	97.28	98.72

The accuracy improvement trend was consistent, rising from 95.33% in the original model to 98.75% with the combined techniques. Recall also improved, increasing from 95.79% to 98.72%, demonstrating enhanced capability in identifying positive samples. F1 scores followed a similar trend, improving from 95.72% to 98.72%, indicating a better balance between precision and recall.

Overall, the combined use of the CLA attention mechanism, DRB, and MoSViT Block significantly enhanced the model's accuracy, recall, and F1 scores, demonstrating that these technologies effectively improved the model's overall performance.

#### 3.3.2 Comparison of different attention mechanisms

To verify the superiority of the CLA attention mechanism proposed in this paper, we conducted systematic comparative experiments. The primary goal was to compare the CLA attention mechanism with other mainstream attention mechanisms to evaluate its impact on model performance. In these experiments, we replaced the attention mechanism in the base model with EMA, CA, SE, ECA, CBAM, and the model's original attention module, respectively. We then compared the performance of these replacements with that of the CLA attention mechanism.

**Figure 10:**
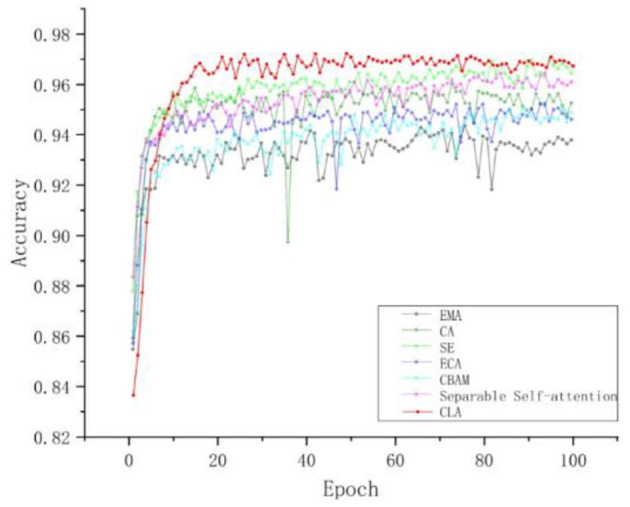
Comparison of experimental accuracy curves of different attention mechanisms. The red curve is the CLA attention mechanism, and the highest accuracy is 97.23%.

To provide a comprehensive evaluation of each attention mechanism's effects, we plotted the accuracy curves. As shown in Figure 10 and Table 7, the accuracy variations of each attention mechanism at different training stages are clearly illustrated. The results indicate that the CLA attention mechanism significantly improves both accuracy and stability, outperforming other attention modules by a substantial margin.

**Table 7:** Comparative test evaluation index data of different attention mechanisms.

**Model**	**Accuracy(%)**	**Precision(%)**	**Recall(%)**	**F1 score(%)**	**Parameter quantity**
EMA	93.17	93.16	93.17	93.00	7,537,268
CA	95.03	95.09	95.03	94.94	3,679,408
CBAM	93.73	93.75	93.73	93.54	3,305,974
ECA	94.33	94.31	94.33	94.21	3,156,173
SE	95.79	95.32	95.33	95.27	1,306,948
Separable self-attention	95.33	95.80	95.79	95.72	4,399,245
CLA	96.24	96.21	96.24	96.11	4,399,245

In summary, comparative experiments have shown that the CLA attention mechanism has significant advantages in improving model performance. These results not only validate the effectiveness of the CLA mechanism in feature extraction and information fusion but also provide new ideas and directions for future model improvement.

#### 3.3.3 Comparative test

To thoroughly evaluate the performance of our enhanced CLA-MoSViT model, we designed a series of comparative experiments. In these experiments, we compared CLA-MoSViT with several prominent deep-learning models, including DenseNet121, Swin Transformer V2, ResNet50, ConvNextV2, Inception-Next-T, GhostNetV2, EfficientNet, MobileNetV2 (Gulzar et al., 2024), and DeiT3. These models represent influential architectures in the field of image recognition, making them ideal for establishing a clear and comprehensive performance benchmark.

In the comparative experiments, we used the same dataset and ensured consistency in the experimental environment to maintain the comparability of results. We evaluated each model based on loss function, accuracy, number of parameters, model size, and other relevant metrics. The experimental results are presented in Supplementary Figures S3– S6 and Table 8.

**Table 8:** Comparative test evaluation index data of different models.

**Model**	**Accuracy(%)**	**Precision(%)**	**Recall(%)**	**F1 score(%)**	**Parameter quantity**	**Single batch inference speed(s)**
Densnet121	98.10	97.44	97.34	97.35	7,978,856	0.9947
Swin Transformer V2	98.44	98.13	97.98	97.92	28,333,468	2.4226
Resnet18	97.45	96.81	96.27	96.24	11,689,512	0.6498
ConvNeXt V2	97.41	96.71	96.25	96.45	27,866,496	1.0658
Inception_Next_T	95.94	94.74	94.43	94.48	28,055,680	1.0022
GhostNet V2	91.32	88.53	87.75	87.93	12,392,698	0.4297
EfficientNet V2	94.55	92.91	92.20	92.17	13,649,388	0.7578
MobileNet V2	84.48	79.61	78.86	78.28	1,968,680	0.2522
Deit3	98.14	97.58	97.25	97.31	22,059,496	0.6252
MobileViT V2	95.33	95.80	95.79	95.72	4,399,245	0.4691
CLA-MoSViT	98.75	98.73	98.72	98.72	7,607,372	0.5964

During the experiment, the CLA-MoSViT model was trained and evaluated meticulously. The outcomes reveal that the model starts to converge after 20 training rounds, and the final value of the loss function stabilizes at approximately 0.001. In contrast, the loss values of other models mostly stabilize at around 0.004. This finding not only demonstrates that the CLA-MoSViT model can achieve an excellent convergence effect within a short training cycle but also showcases its remarkable advantages in training efficiency. Generally speaking, the CLA-MoSViT model performs outstandingly, featuring high training efficiency and superior performance.

Comparative data highlights that CLA-MoSViT excels across key metrics, including average accuracy, precision, recall, F1 score, and parameter count. Notably, CLA-MoSViT achieves an outstanding average accuracy of 98.75%, outperforming all other models, which demonstrates its effectiveness in correctly classifying instances in the dataset. Furthermore, with a precision score of 98.73%, CLA-MoSViT leads in ensuring that positive classifications are highly likely to be correct. The recall score stands at 98.72%, also the highest among the models, indicating its ability to accurately identify a high proportion of actual positives. The F1 score of 98.72% underscores a balanced performance between precision and recall.

Impressively, despite its superior performance, CLA-MoSViT boasts significantly fewer parameters−7,607,372—compared to models like Swin Transformer V2, ConvNeXt V2, and DeiT3, making it more efficient in terms of computational resources. Excellent inference speed in high-precision models −0.5964s per batch. These results further validate the effectiveness of our improved strategy and indicate the potential superiority of the CLA-MoSViT model in practical applications.

Through this series of comparative experiments, we not only verify the performance of the CLA-MoSViT model but also provide a valuable reference and comparison benchmark for future research. We believe that with further optimization and improvement, the CLA-MoSViT model will play a more significant role in areas such as image recognition.

#### 3.3.4 Thermal map comparison test

**Figure 11:**
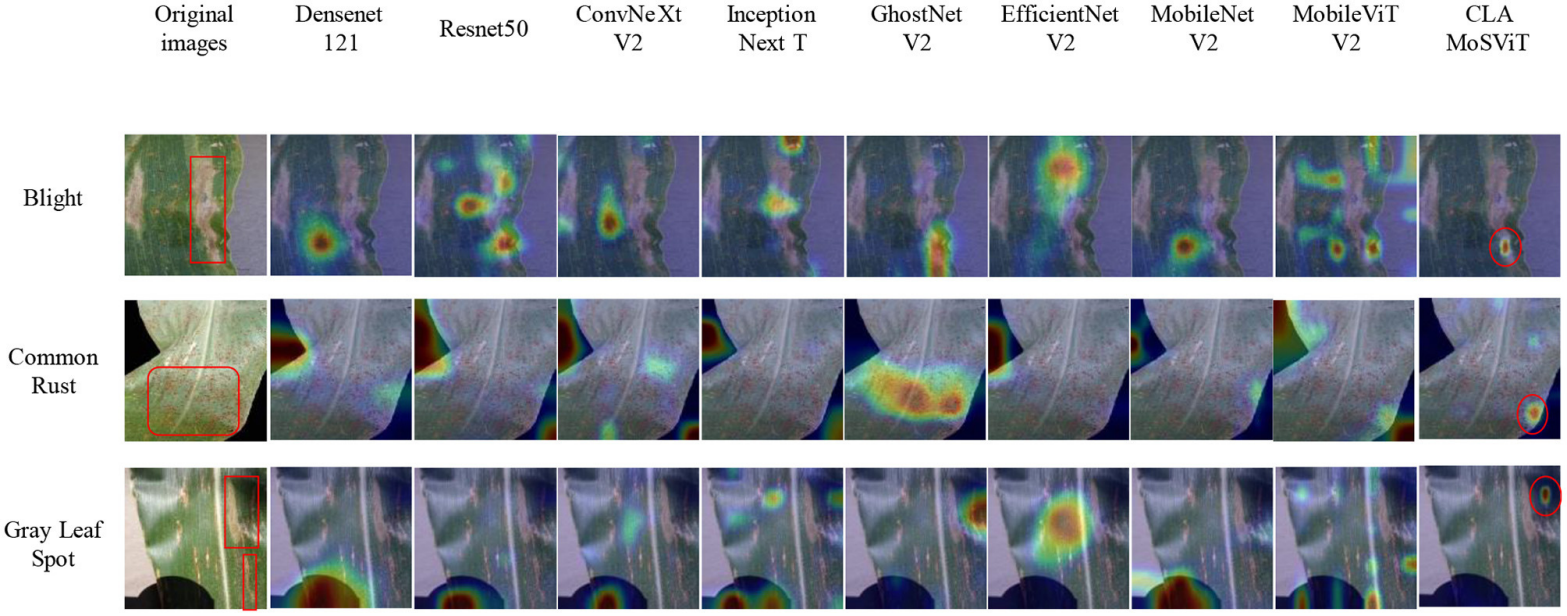
Heat maps of different models.

To further verify the superiority of the CLA-MoSViT algorithm, we adopted the thermal map method to demonstrate the advantages of the model in feature extraction and area of concern. By comparing the heat maps of CLA-MoSViT and the baseline model on different test images, we were able to visually observe the accuracy and effectiveness of the model in identifying key areas. The thermal map results are shown in Figure 11.

We can see the significant advantages of CLA-MoSViT in focusing on important features and suppressing interference factors, which are essential for detection in complex environments. The model's focus area is more precise and aligns closely with our ideas. These results not only validate the effectiveness of the improved algorithm but also further demonstrate the superior performance of CLA-MoSViT in practical applications.

#### 3.3.5 Confusion matrix experiment

To analyze the performance of our enhanced CLA-MoSViT model in greater detail and to better understand its class recognition capabilities, we conducted a confusion matrix experiment. Similar to the previous comparative tests, we included DenseNet121, Swin Transformer V2, ResNet50, ConvNeXt V2, Inception-Next-T, GhostNetV2, EfficientNet, MobileNetV2, and DeiT3 as comparison models.

A confusion matrix is a valuable tool for visualizing how a model's true classifications compare with its predicted classifications across different categories. It allows us to see which categories the model excels in and where it may make errors or experience confusion. In this experiment, we recorded the prediction results of each model on the test set in detail and generated the corresponding confusion matrix graphs. The results are illustrated in the figures.

By comparing the confusion matrices of each model, we observed that the CLA-MoSViT model generally demonstrates higher recognition accuracy across most categories and a lower misjudgment rate. This further validates the effectiveness of the CLA-MoSViT model in image recognition tasks. The experimental results of the confusion matrix are shown in Supplementary Figures S9, S10.

#### 3.3.6 Test experiments with different data sets

**Figure 12:**
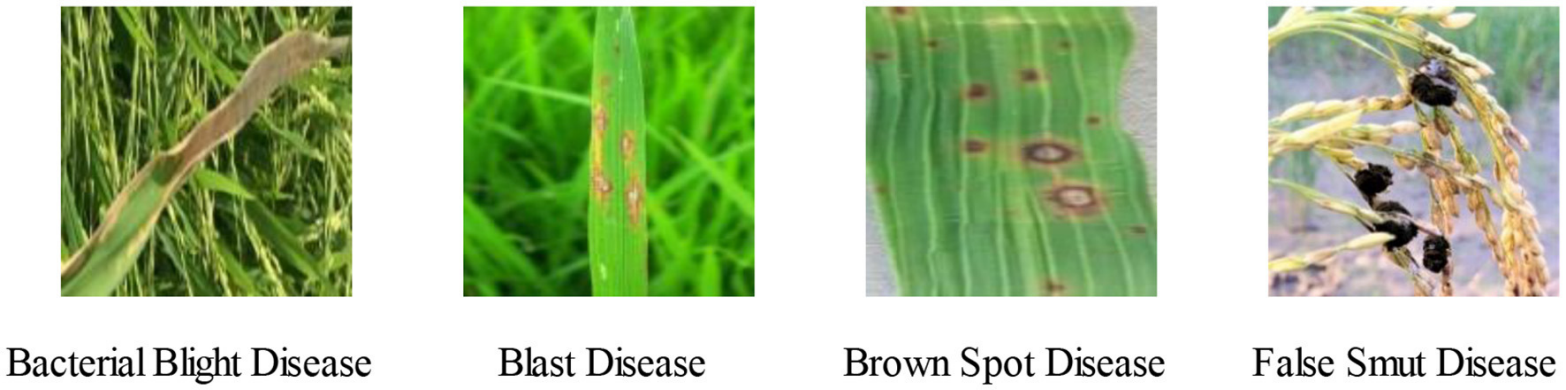
Representative images of the rice dataset.

To further verify the generality of the CLA-MoSViT algorithm and whether the model can efficiently capture key features with a small number of samples, we conducted a test experiment with different data sets. A dataset of rice diseases was used in this experiment. The images of each species are shown in Figure 12, and the number and species of images in the dataset are shown in Table 9. Without any enhancement of the data, all models are tested directly on the original data set, and the performance of each model is compared.

**Table 9:** Number of images in rice small sample dataset.

**Type of disease**	**Image quantity**	**Training set size**	**Test set size**
Bacterial blight disease	50	40	10
Blast disease	50	40	10
Brown spot disease	50	40	10
False smut disease	50	40	10

The comparison between the detection results and other mainstream models is shown in Supplementary Figure S1 and Table 10.

**Table 10:** Test evaluation index data of small sample datasets of different models.

**Model**	**Accuracy(%)**	**Precision(%)**	**Recall(%)**	**F1 score(%)**	**Parameter quantity**
Densnet121	78.08	78.26	74.66	75.12	7,978,856
Swin Transformer V2	60.43	56.80	61.02	57.76	28,333,468
Resnet18	48.83	37.18	42.66	38.60	11,689,512
ConvNeXt V2	78.30	75.16	74.51	74.17	27,866,496
Inception_Next_T	70.20	70.12	69.71	67.94	28,055,680
GhostNet V2	60.10	60.06	62.71	57.98	12,392,698
EfficientNet V2	55.48	54.91	57.00.	52.38	13,649,388
MobileNet V2	49.13	51.77	49.93	44.58	1,968,680
Deit3	67.43	68.17	65.58	63.83	22,059,496
MobileViT V2	81.40	82.49	81.75	80.82	4,399,245
CLA-MoSViT	86.08	85.89	85.05	84.86	7,607,372

The experimental results showed that CLA-MoSViT achieved excellent results on the small sample data set of rice diseases. First of all, it shows that the CLA-MoSViT model has a strong generalization and the model solves the problem of overfitting. Secondly, the model still shows good performance when the number of samples is very small, the image quality is not high and some features are similar, which not only shows the superiority of the model but also shows that our “attention theory” is correct and verifiable.

**Figure 13:**
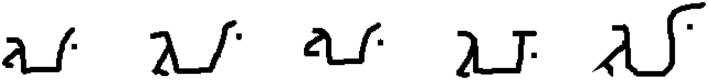
The Alphabet_of_the_Magi font is written by different people.

To further validate the model's performance on few-shot learning tasks, we conducted experiments using the Omniglot benchmark dataset (Lake et al., 2015). As a specialized image dataset for few-shot character recognition, Omniglot contains 1,623 unique characters from 50 distinct writing systems, each rendered by 20 different individuals. Figure 13 illustrates representative handwriting samples of the Alphabet_of_the_Magi script. This evaluation specifically examines the model's capability in cross-linguistic^*^ few-shot classification scenarios. Supplementary Figures S7, S8 show the test results.

The experimental results demonstrate that the model achieves exceptional performance in both convergence speed and final classification accuracy. These findings suggest that the proposed architecture deserves further investigation in the field of few-shot learning.

## 4 Conclusion

This study aims to improve the classification performance of models in complex environments. By introducing the CLA attention mechanism, DRB module, MoSViT Block, and LeakyReLU6 activation function, we have significantly enhanced the learning efficiency and accuracy of the model. The results of our experiments demonstrate that the enhanced focus and accuracy of the model significantly improve classification performance in complex environments. The model effectively captures only the most important features in an image, thereby increasing learning efficiency.

Specifically, the CLA attention mechanism proposed in this paper excels in gathering global information and focusing attention. In the attention mechanism comparison experiments, it achieves an accuracy rate of over 96%, which is notably higher than other attention mechanisms. The DRB module and MoSViT Block designed in this study substantially enhance model performance with only a minimal increase in parameters. Ablation experiments show that these components improve the average accuracy of the original model by 0.7% and 1.95%, respectively.

The LeakyReLU6 activation function effectively prevents model overfitting and improves computational stability, maintaining superior performance across different datasets. Due to its high learning efficiency, the model performs well even with small sample sizes. This further supports our findings and may guide future research directions.

However, despite the significant achievements of this study, there are still some limitations to our approach. For example, the convergence speed and accuracy of the model with a small number of samples need to be improved. In future studies, we plan to explore the potential of precision attention theory in the field of small-sample detection.

## Data Availability

The original contributions presented in the study are included in the article/Supplementary material, further inquiries can be directed to the corresponding author.
