# Supplementary figures and images for: MoSViT: a lightweight vision transformer framework for efficient disease detection via precision attention mechanism
Source: Front Artif Intell. 2025 Mar 26;8:1498025. doi: 10.3389/frai.2025.1498025 (PMC11979205; doi:10.3389/frai.2025.1498025)

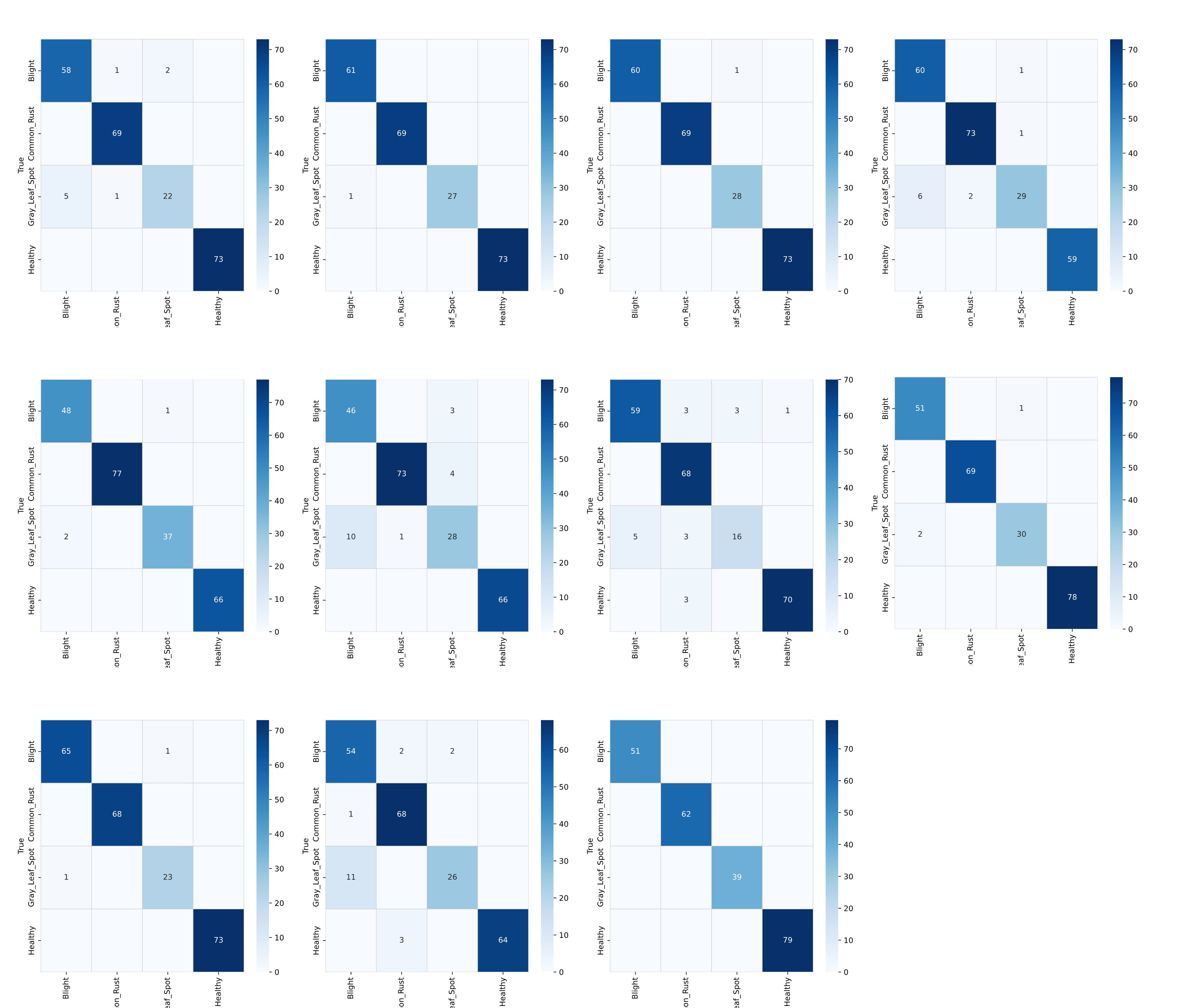

Supplement: Supplementary file 1 [file Image_1.tif]

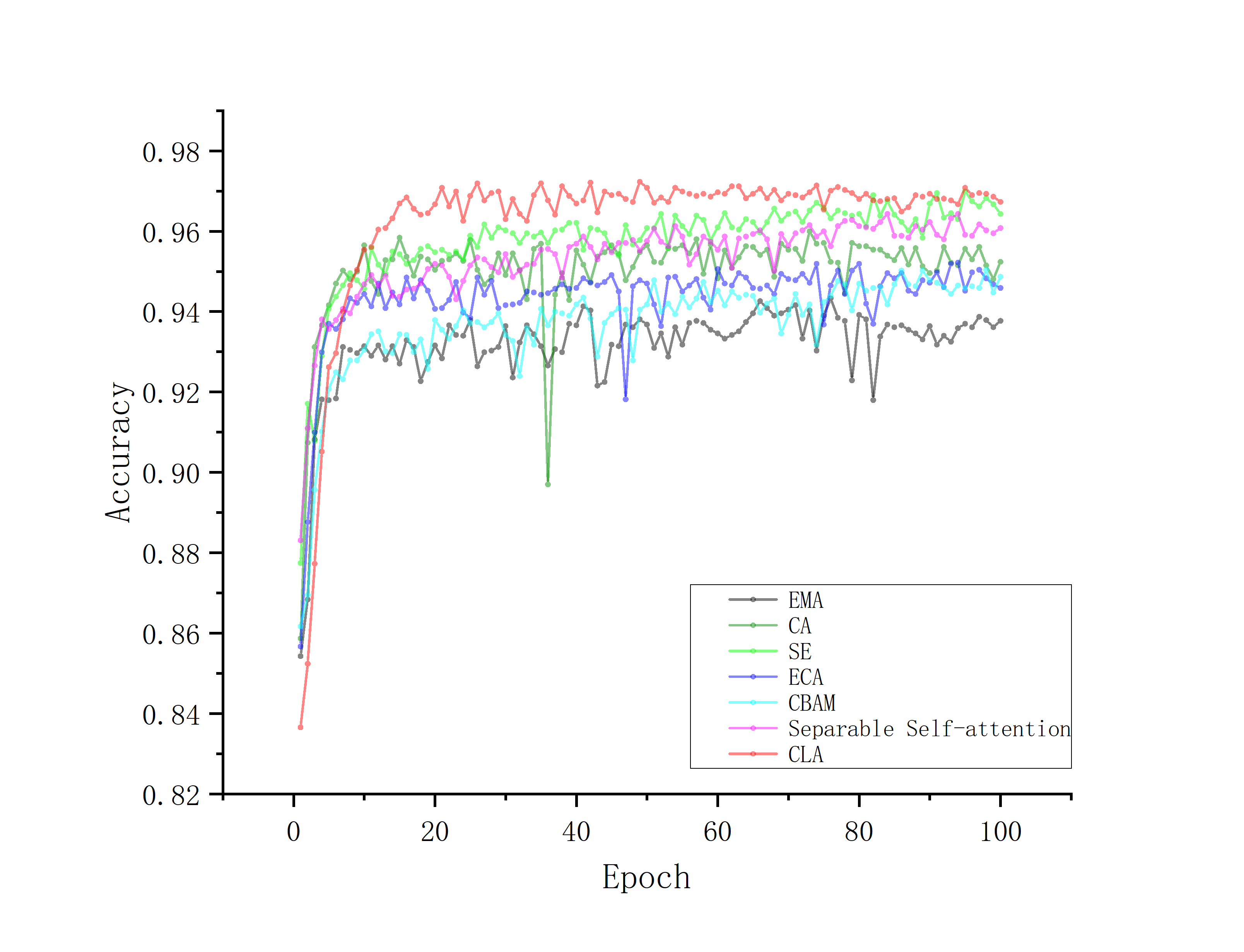

Supplement: Supplementary file 2 [file Image_2.tif]

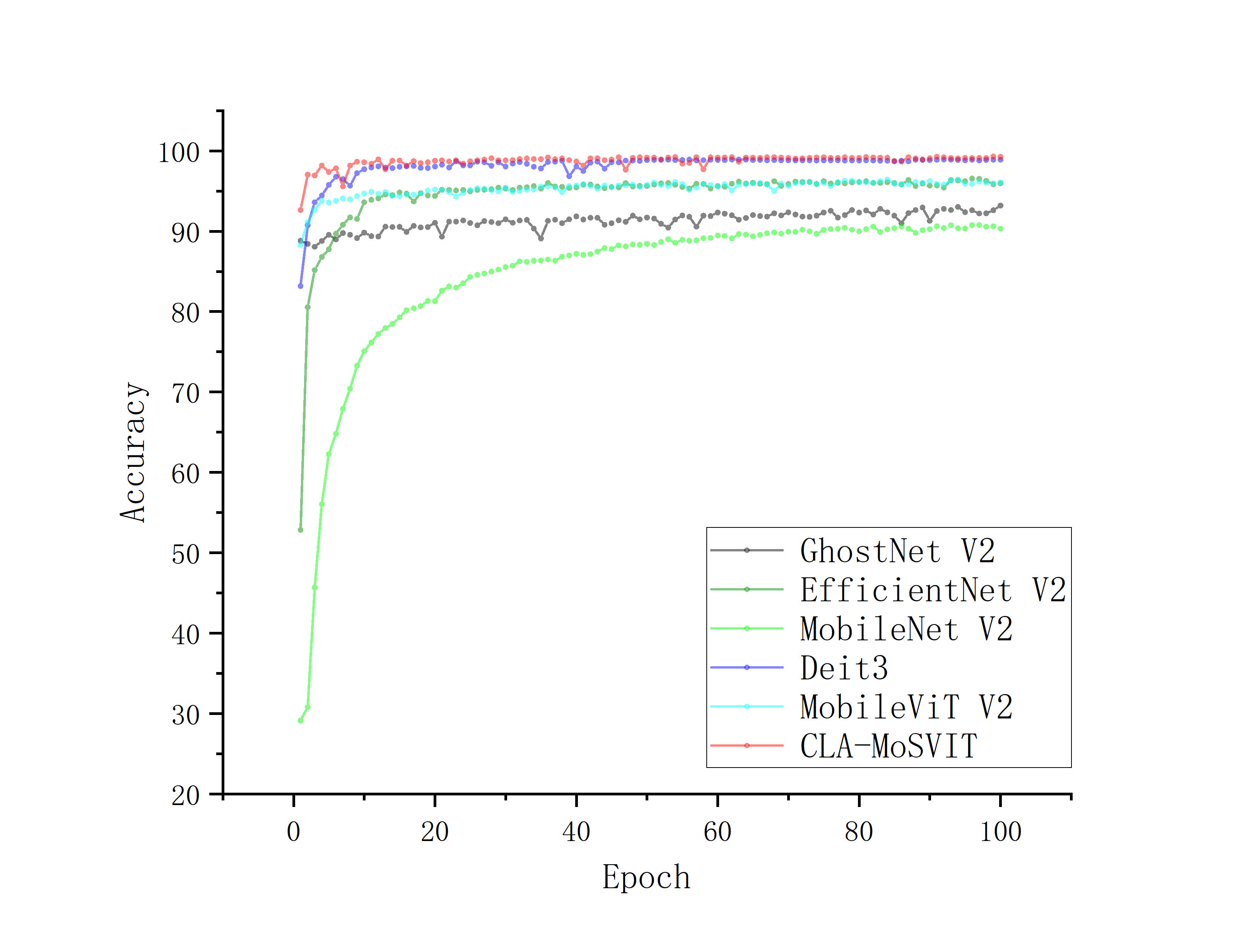

Supplement: Supplementary file 3 [file Image_3.tif]

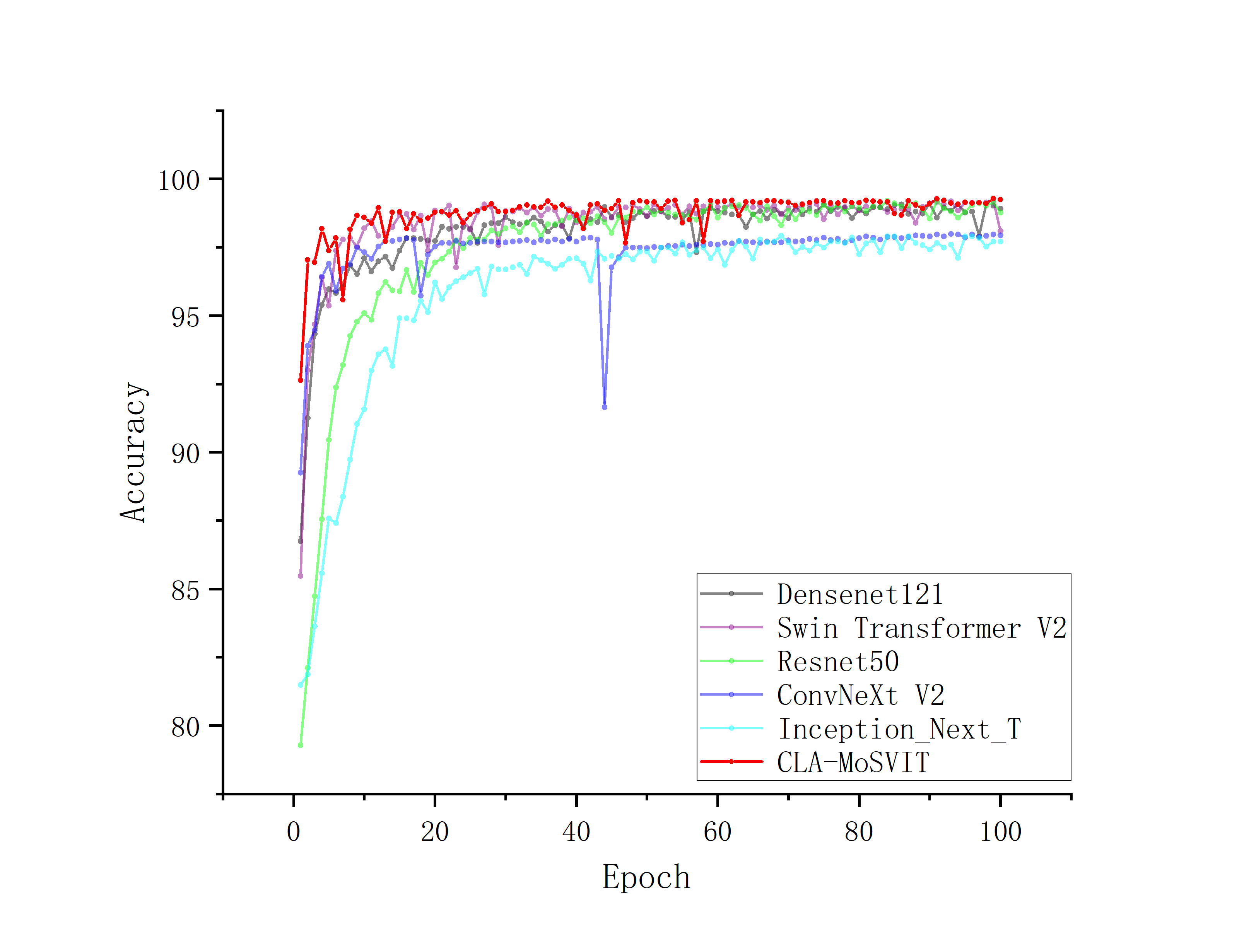

Supplement: Supplementary file 4 [file Image_4.tif]

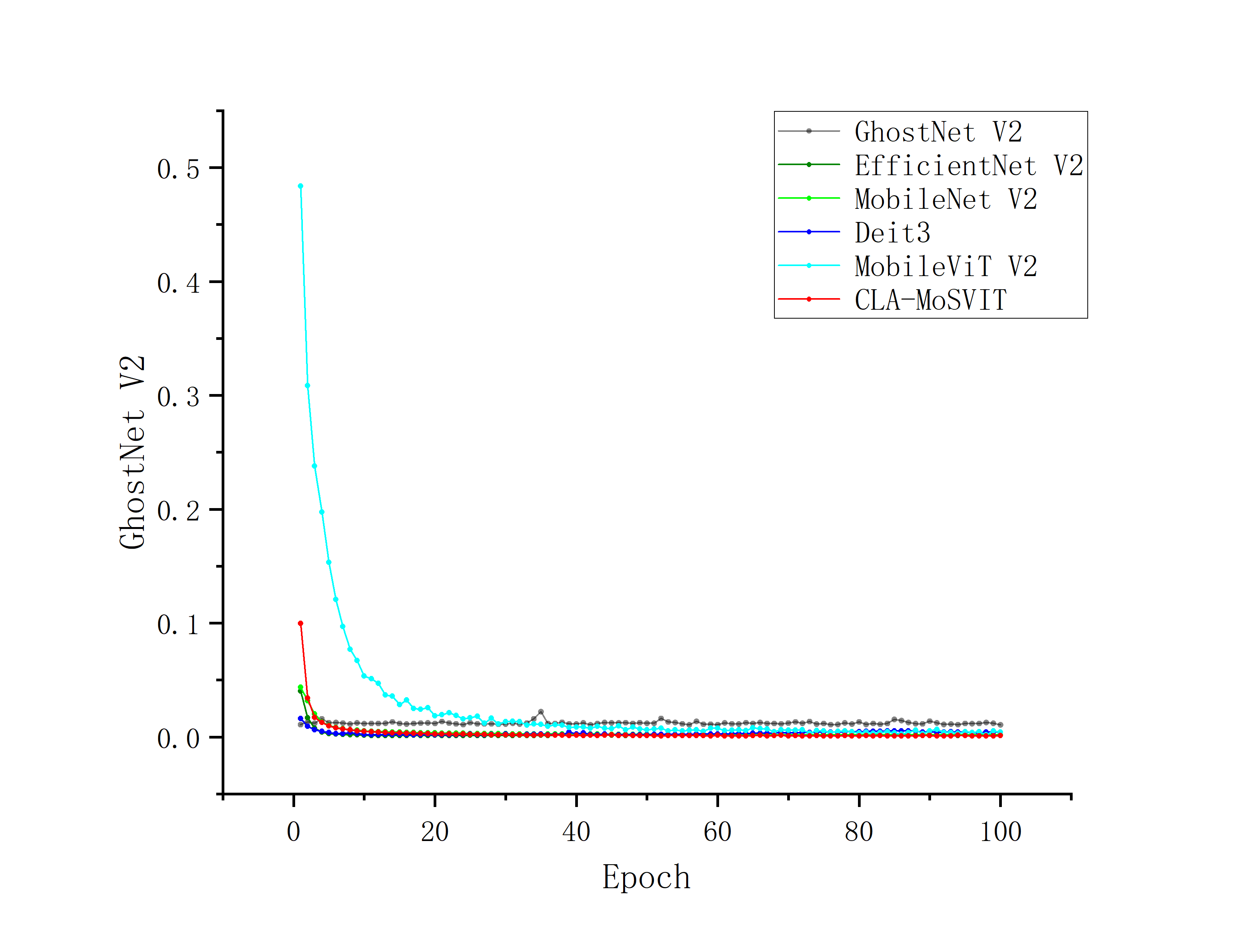

Supplement: Supplementary file 5 [file Image_5.tif]

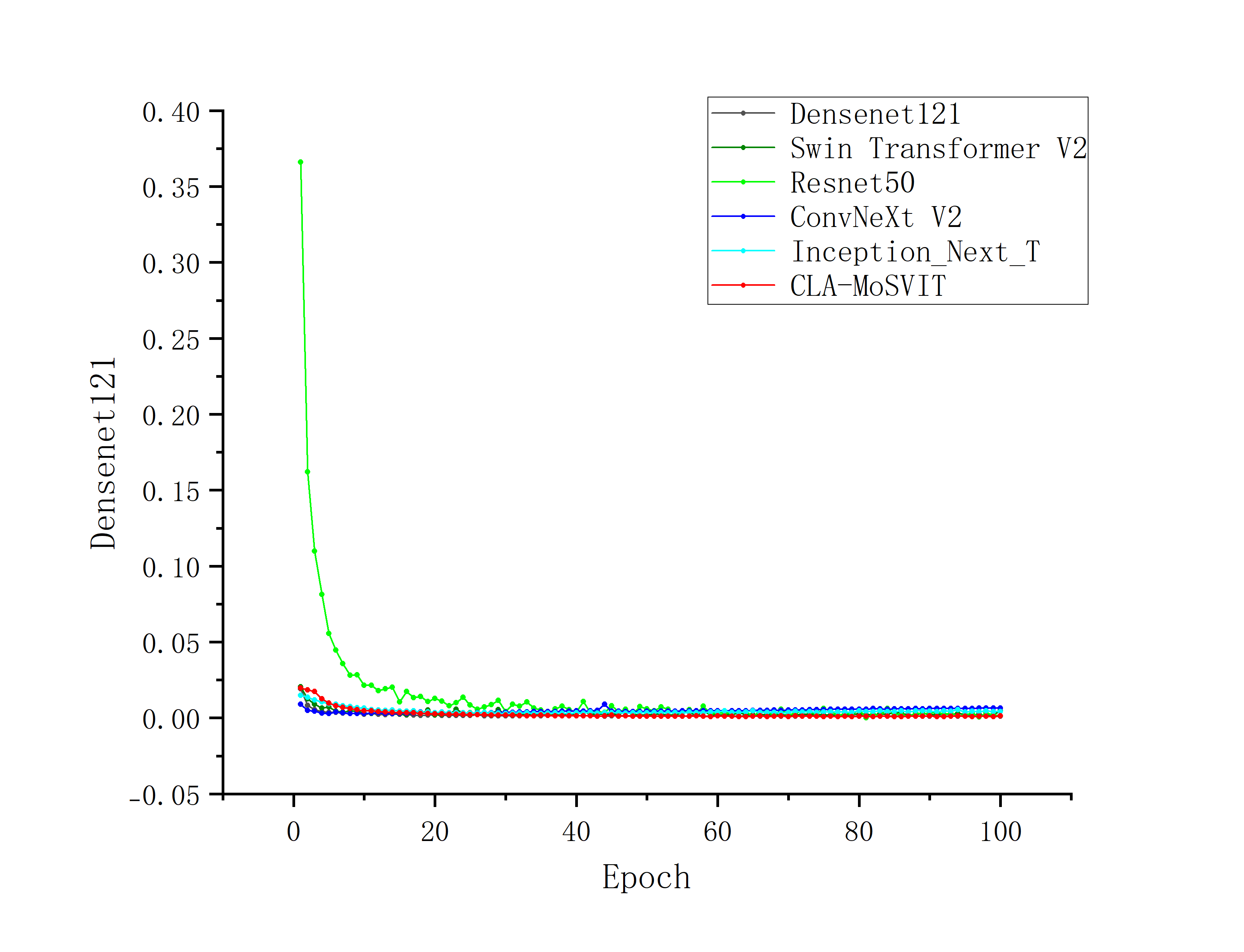

Supplement: Supplementary file 6 [file Image_6.tif]

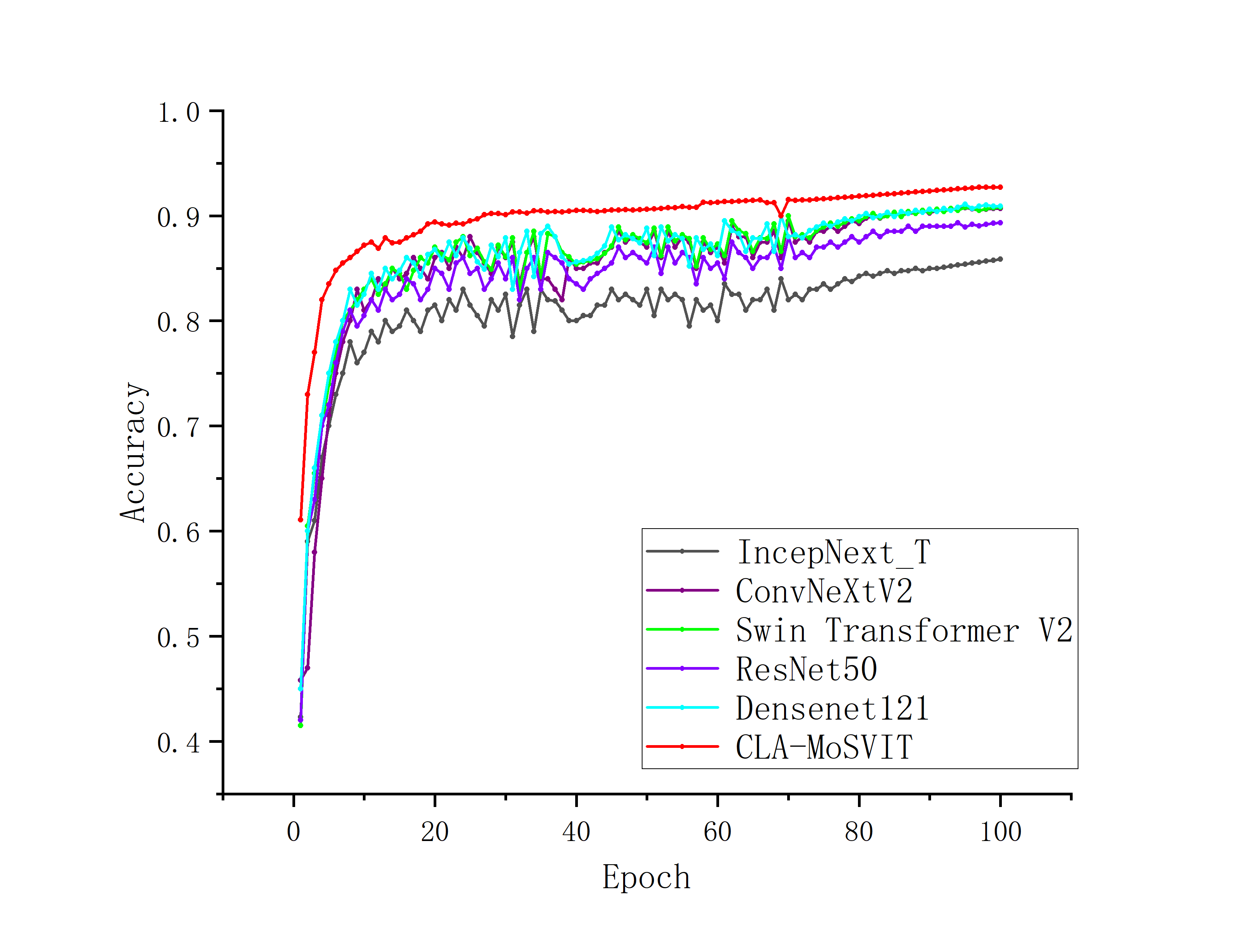

Supplement: Supplementary file 7 [file Image_7.tif]

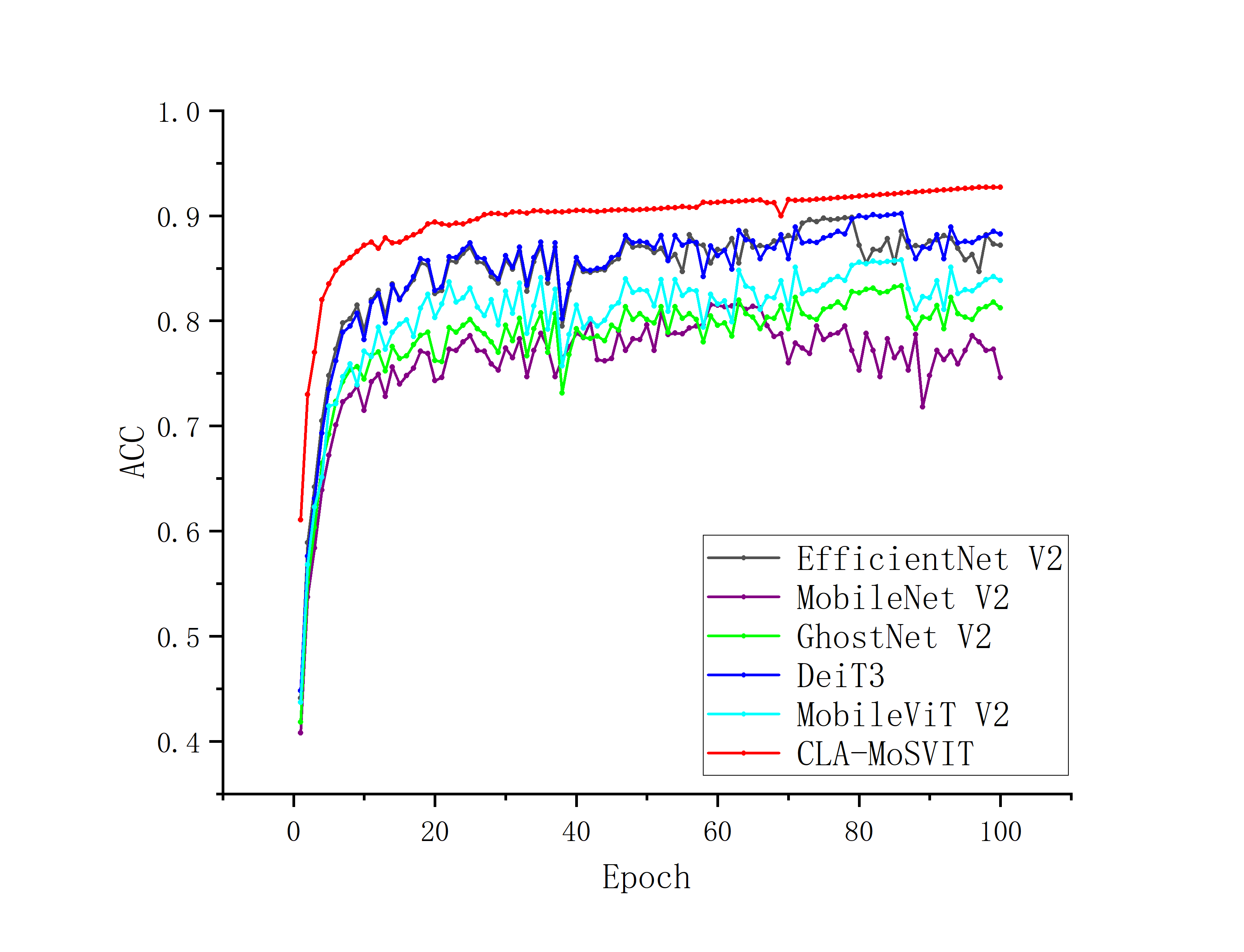

Supplement: Supplementary file 8 [file Image_8.tif]

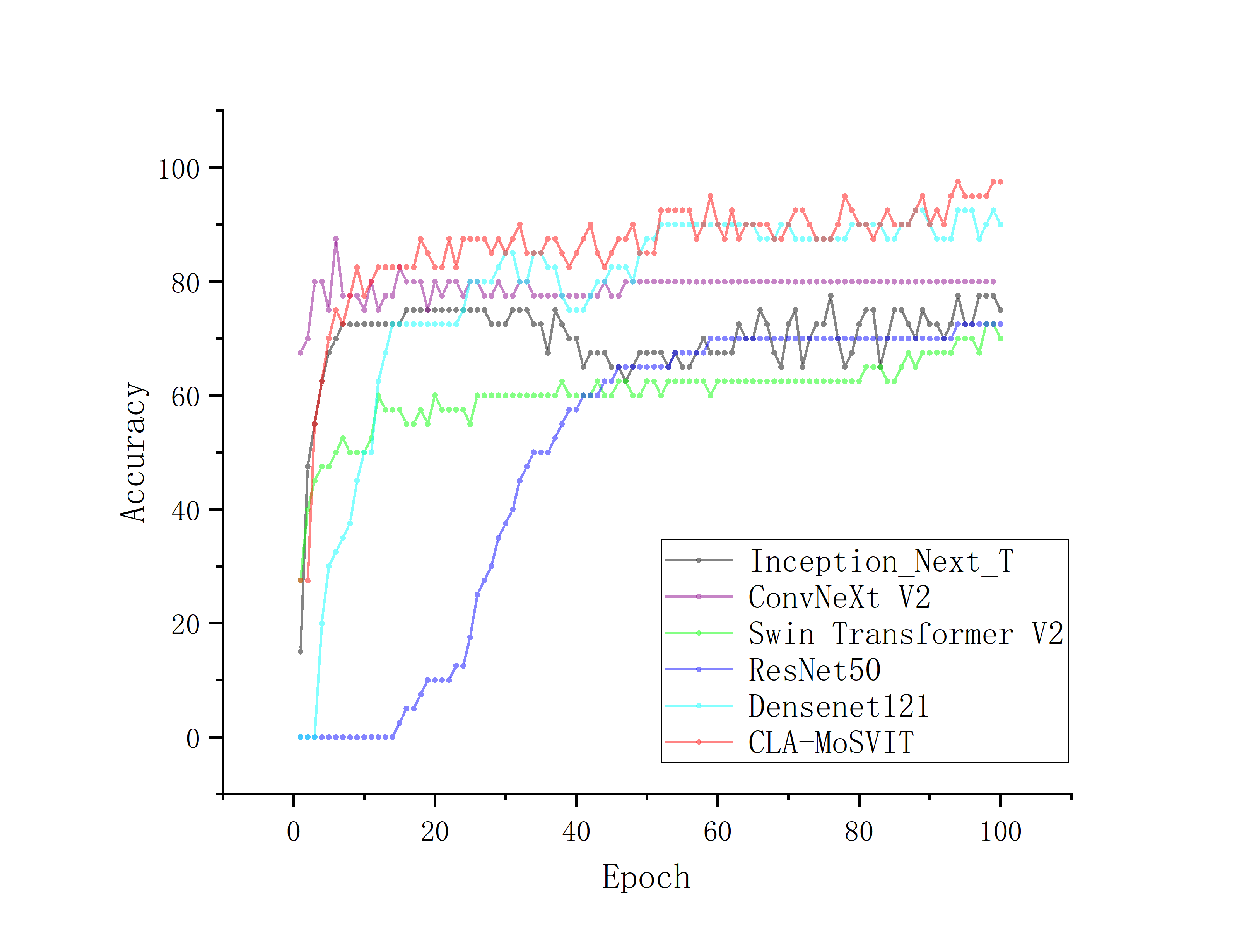

Supplement: Supplementary file 9 [file Image_9.tif]

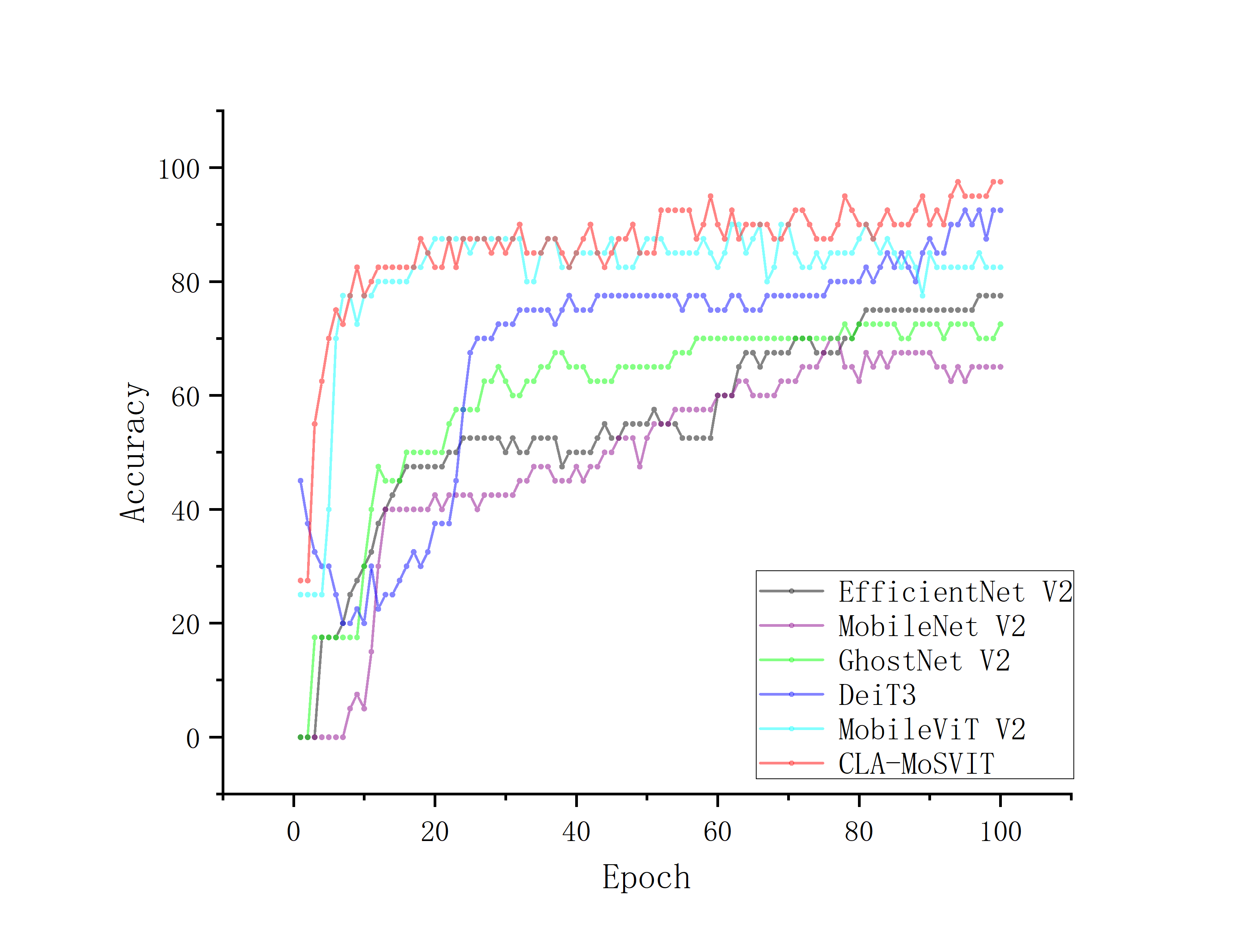

Supplement: Supplementary file 10 [file Image_10.tif]
